# Japanese Encephalitis Virus Genotype 5 Infectious Clone and Reporter System for Antiviral Evaluation

**DOI:** 10.1002/jmv.70608

**Published:** 2025-09-16

**Authors:** Jae‐Yeon Park, Wataru Kamitani, Hyun‐Jin Shin, Hye‐Mi Lee

**Affiliations:** ^1^ College of Veterinary Medicine Chungnam National University Daejeon South Korea; ^2^ Department of Infectious Diseases and Host Defense Gunma University Graduate School of Medicine Gunma Japan

**Keywords:** antiviral screening assay, genotype 5, infectious clone, Japanese encephalitis virus, Reporter virus

## Abstract

The recent emergence of Japanese encephalitis virus genotype 5 (JEV5) has raised concerns regarding limited vaccine efficacy. Here, we report the construction of a full‐length infectious cDNA clone of JEV5 using a bacterial artificial chromosome (BAC) system, based on a South Korea isolate, K15P38 strain. The resulting clone enabled recovery of replication‐competent virus and was genetically distinguishable from the parental strain via engineered silent mutations. To extend its application, we introduced nanoluciferase and enhanced green fluorescent protein into the viral genome, generating reporter viruses suitable for replication tracking. Both reporter viruses supported real‐time quantification of viral replication and demonstrated utility in antiviral compound screening. Antiviral evaluation using the nucleoside analog NITD008 yielded EC₅₀ values consistent between reporter and classical plaque assays. These findings provide a reverse genetics platform for studying JEV5 and suggest applicability for genotype‐specific vaccine and antiviral research.

## 1 Introduction

Japanese encephalitis virus (JEV), a mosquito‐borne flavivirus, is the etiological agent of Japanese encephalitis (JE), an inflammatory neurological disease affecting humans across Asia and the Western Pacific [1, 2, 3, 4]. In endemic regions, JE predominantly impacts children younger than 15 years of age, although travel‐associated JE can occur in individuals of any age who lack pre‐existing immunity to JEV [5]. The transmission of JEV occurs through an enzootic cycle involving *Culex* mosquitoes and amplifying hosts, including ardeid birds and pigs [6]. However, humans serve as dead‐end hosts, as viremia levels are typically insufficient to facilitate onward transmission to mosquito vectors [7].

JEV, a member of the *Flaviviridae* family and classified under the *Ortho*f*lavivirus* genus, possesses a single‐stranded, positive‐sense RNA genome of approximately 11 kb, enclosed within a lipid envelope. The genome comprises a single open reading frame (ORF) bounded by 5′ and 3′ untranslated regions (UTRs). The ORF is translated into a polyprotein precursor, which undergoes proteolytic processing to yield three structural (capsid [C], premembrane [prM], and envelope [E]) and seven nonstructural (NS1‐NS2A‐NS2B‐NS3‐NS4A‐NS4B‐NS5) proteins [8, 9]. The C protein forms the highly ordered nucleocapsid that packages and protects the viral RNA genome [10]. This nucleocapsid is surrounded by a host‐derived lipid bilayer containing two envelope‐associated proteins, prM and E [11]. The prM protein assists in the proper folding and stabilization of the E protein during virion assembly and is subsequently cleaved during the maturation of infectious particles [11]. The E protein, which forms homodimers on the viral surface before maturation, mediates host cell attachment and membrane fusion, thereby serving as a major determinant of viral tropism and a key target for neutralizing antibodies [12]. Proteolytic processing of the polyprotein is mediated by both host and viral proteases, with the NS2B‐NS3 complex playing a central role in generating functional proteins required for viral replication [13].

Molecular phylogenetic analyses of the E protein sequence has divided JEV into five genotypes (JEV1‐5) [14]. JEV5, represented by the Muar strain, was initially identified in a patient with encephalitis in Malaysia [15]. Early phylogenetic analyses classified the Muar strain into a distinct JEV5 lineage, and subsequent evolutionary studies suggested that JEV5 may represent the most ancestral lineage from which other JEV genotypes have diverged [16]. A follow‐up whole‐genome‐based phylogenetic analysis further supported this classification, indicating JEV5 as the genotype closest to the common ancestor, with JEV4, JEV3, JEV2, and JEV1 diverging sequentially [17]. Since JEV4 and JEV5 are considered to be evolutionarily ancestral genotypes, more recent isolates have predominantly consisted of JEV3 and JEV1 [18]. While JEV3 was initially prevalent in early surveillance efforts, it was gradually supplanted by JEV1 across most endemic regions [19]. Recently, the increasing detection of JEV5 has raised concerns regarding its re‐emergence and potential broader distribution [20, 21]. Accumulating evidence indicates that JEV5 is increasingly prevalent in some regions, highlighting the ongoing genotype shifts that might affect both surveillance and vaccine efficacy [22].

Although JEV is classified as a single serotype [14], it comprises five distinct genotypes (JEV1–5), presenting significant challenges for JE control and prevention [23]. Notably, JEV5 is highly pathogenic and causes early viremia and central nervous system (CNS) invasion in animal models. However, its epidemiological characteristics and global distribution remain poorly understood [24]. Previous studies had indicated that neutralizing antibodies elicited by genotype‐specific JEV vaccines, such as those from JEV3‐ or JEV5‐based strains, exhibit limited cross‐neutralization efficacy against heterologous genotypes [25]. Therefore, evaluation of the protective breadth of existing JEV vaccines and the development of genotype‐specific or broadly protective vaccine candidates through further studies focused on their immunogenicity and cross‐reactivity are urgently required [26].

In parallel with vaccine development, the discovery of effective antiviral compounds remains a critical approach for controlling JEV infections, particularly in endemic regions [27]. Traditional antiviral screening methods, such as the plaque reduction assay (PRA), have long been used to evaluate the antiviral efficacy of candidate compounds by quantifying plaque formation in infected cell cultures [28, 29]. Although PRA is widely used for its reliability in measuring antiviral activity, it is time‐consuming, labor‐intensive, and requires prolonged incubation periods for plaque formation [30]. To address these limitations, modern approaches have turned to high‐throughput screening (HTS) platforms, which utilize reporter viruses that are genetically engineered to express fluorescent or luminescent reporter markers [31, 32]. These technologies allow the rapid and automated quantification of viral replication, thereby significantly accelerating the antiviral drug screening process.

In this study, we developed a cDNA‐based infectious clone of a JEV5 strain (K15P38) isolated in South Korea using a bacterial artificial chromosome (BAC) system. The infectious clone enabled the characterization of the genetic and phenotypic features of JEV5, providing insights into replication kinetics, cytopathic effects, and pathogenic potential. To explore genotype‐related differences in viral behavior, the JEV5 clone was directly compared to a previously developed JEV3 clone, and virological properties, including plaque morphology, growth kinetics, and neurovirulence, were systematically evaluated using a mouse model. Furthermore, to expand the utility of our JEV clones, we engineered reporter virus constructs that incorporated nanoluciferase (NLuc) and enhanced green fluorescent protein (EGFP) genes. The constructs were designed to facilitate high‐throughput antiviral compound screening by enabling rapid and quantitative assessments of viral replication. Our findings supported the applicability of this reporter virus system for antiviral screening and broader virological investigations, highlighting the utility of the reporter virus system as a flexible and efficient platform for further JEV research.

## 2 Materials and Methods

### 2.1 Cells and Viruses

African green monkey kidney cell lines (Vero; CCL‐81, ATCC) were maintained in Minimum Essential Media (MEM; Welgene, South Korea) supplemented with 10 mM HEPES (Gibco, NY, USA), 1**×** Antibiotic‐Antimycotic (Gibco), and 10% fetal bovine serum (FBS; Gibco, MA, USA) at 37°C in a 5% CO_2_ incubator. JEV5 K15P38 strain (GenBank: MK541529) was obtained from the Korea Disease Control and Prevention Agency (KDCA). The previously reported JEV3 infectious clone (JYJEV3) was used in this study [33]. JEV stocks and infectious clones were prepared on Vero cells.

### 2.2 Generation of a Full‐Length Infectious cDNA Clone of JEV5

**Figure 1:**
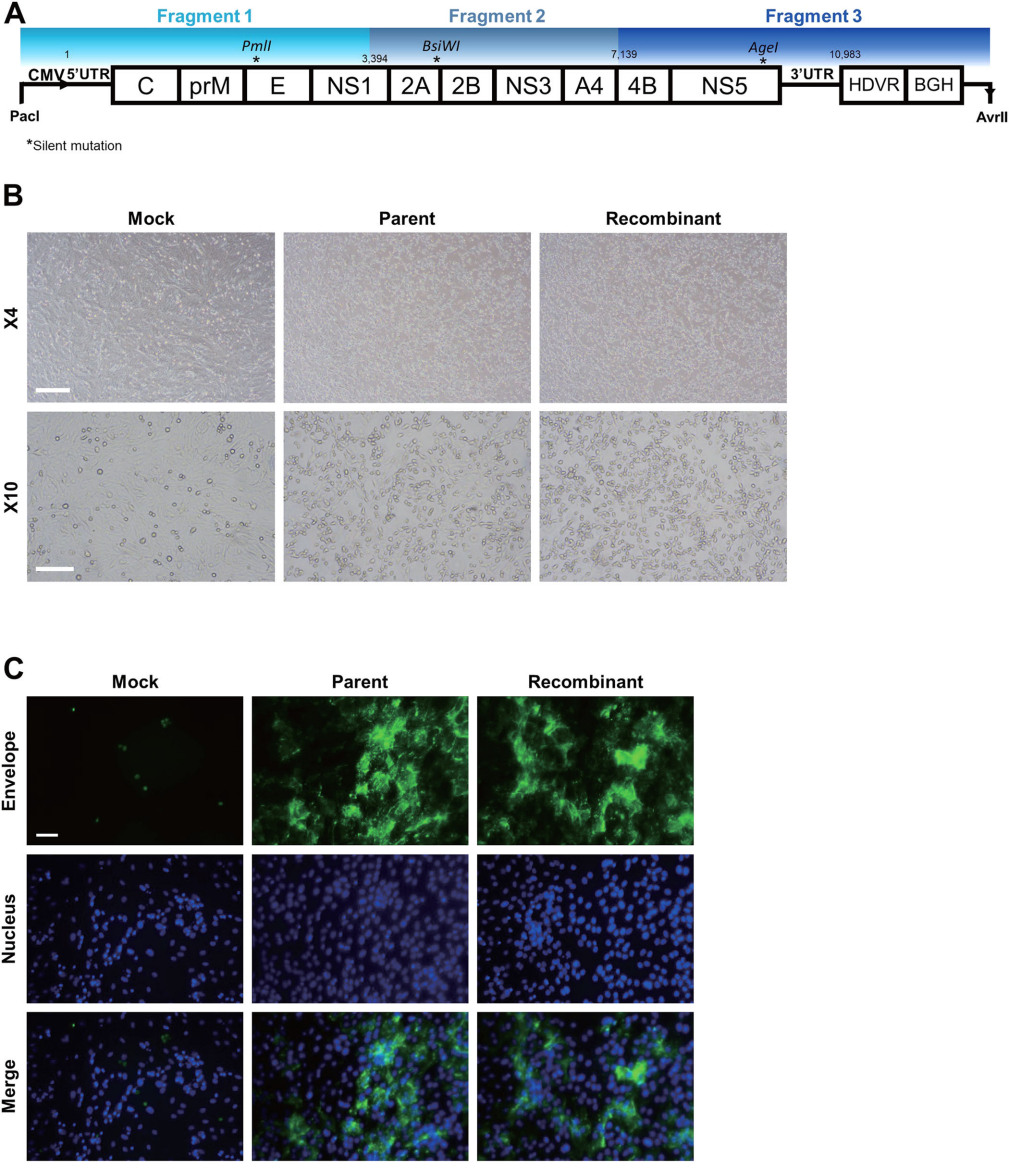
Construction and rescue of a full‐length infectious cDNA clone of JYJEV5. (A) Schematic diagram of pBAC‐JYJEV5 construction, comprising three fragments (F1–F3) assembled under the control of a CMV promoter, with an HDVr and BGH polyadenylation signal. (B) CPE in Vero cells transfected with the pBAC‐JYJEV5 plasmid (5 dpt) or infected with parental JEV5 (MOI = 0.01, 4 dpi), shown at ×4 (upper panels) and ×10 (lower panels) magnifications. Scale bar, 200, 400 μm. (C) Immunofluorescence staining of viral E protein (green) in infected Vero cells at 96 hpi. Cell nuclei were stained with Hoechst 333258 (blue). Scale bar, 200 μm. Parent, parent JEV5; Recombinant, JYJEV5; day‐post transfection (dpt). Data represent at least three independent experiments.

Viral RNA was extracted from JEV5 stocks using the Ribospin vRD kit (Geneall, South Korea) and cDNA synthesis was performed using the TOPscript RT DryMIX dT18/dN6 plus (Enzynomics, South Korea). The full‐length JEV genome was divided into nine fragments, which were individually amplified using virus‐specific primers (Supporting Information S1: Table 1). The primers were designed based on the previously published K15P38 sequence, and the extreme 5' and 3' UTR termini of the genome were inferred from other JEV G5 reference sequences. Each fragment was cloned into the pGEMT Easy vector (Promega, WI, USA). Using the In‐Fusion Snap Assembly Master Mix kit (Takara, Japan), the nine fragments were assembled into three larger fragments comprising JEV5 fragment 1 (A + B + C), fragment 2 (D + E + F), and fragment 3 (G + H + I), respectively. These three large fragments were subsequently cloned into pBAC vectors using the In‐Fusion protocol. The final plasmid contained the full‐length cDNA of JEV5 under the control of a CMV promoter, with a hepatitis delta virus ribozyme (HDVr) sequence positioned downstream at the 3' end (Figure 1A).

### 2.3 Sequence Confirmation of Parental and Recombinant Virus

To confirm the identity and genetic stability of parental and recombinant virus, viral RNA was extracted from samples collected across multiple passages. PCR amplification targeting the E, NS2A, and NS5 regions was performed using primers listed in Supporting Information S1: Table 2, designed based on the JEV5 strain K15P38. PCR products were subjected to restriction enzyme digestion using *PmlI*, *BsiWI*, and *AgeI* (all from New England Biolabs, MA, USA). Additionally, amplicons were cloned into the pGEM‐T Easy vector (Promega) and analyzed by Sanger sequencing. Full‐length genome sequencing was also performed on the recombinant virus at passage 20 to assess long‐term genetic stability.

### 2.4 Construction of pBAC‐JYJEV5‐Based Reporter Viruses

Reporter gene‐expressing JEV5 clones were generated by inserting a reporter gene cassette containing the 5′ UTR‐C38, reporter gene‐2A, and scrambled capsid‐prME into the pBAC‐JYJEV5 plasmid vector. The cassette was divided into three fragments—5′ UTR‐C38, reporter gene‐2A, and scrambled capsid‐prME—which were amplified by PCR using the primers listed in Supporting Information S1: Table 4. These three fragments were then assembled into a single cassette through the overlapping PCR method. The resulting cassette was inserted into the linearized pBAC‐JYJEV5 plasmid, which had been digested with the restriction enzymes *PmeI* (New England Biolabs) and *PmlI*. Cloning of the reporter gene cassettes into the linearized plasmid was performed using the In‐Fusion cloning method, following the manufacturer's protocol (Supporting Information S1: Figure 4).

### 2.5 Transfection of the pBAC‐JYJEV5 cDNA Clone Into Vero Cells

The plasmid pBAC‐JYJEV5, containing the full‐length cDNA of JEV5, was amplified in *E. coli* HST08 premium (Takara) and purified using the NucleoBond Xtra Maxi (Machery Nagel, Germany) according to the manufacturer's instructions. The reporter gene constructs (pBAC‐JYJEV5‐NLuc and pBAC‐JYJEV5‐EGFP) were prepared and purified in the same manner. Vero cells were seeded in a six‐well plate, and the transfection mixture was prepared using 1 mL of Opti‐MEM medium (Gibco) containing TransIT‐LT1 (Mirus, WI, USA) and 6 μg of plasmid DNA (either pBAC‐JYJEV5 or reporter gene constructs) for 4 h, following the manufacturer's protocol. The transfection medium was removed and replaced with 2 mL of growth medium.

### 2.6 Amplification and Titration of Parent and Recombinant Viruses From Cell Supernatants

Cell culture supernatants were serially passaged in Vero cells to amplify parental and recombinant viruses (JYJEV5, JYJEV5‐NLuc, and JYJEV5‐EGFP). Briefly, Vero cells were inoculated with supernatants derived from transfected cells and harvested when approximately 80% of the cells exhibited cytopathic effect (CPE). CPE was consistently observed in cells infected with all viruses, including parent and recombinant viruses. The collected samples underwent two freeze‐thaw cycles and were centrifuged at 6000*g* for 15 min to obtain cell‐free supernatants. For subsequent passages, the clarified supernatants were diluted in fresh medium and used to inoculate new Vero cells. Harvested samples were stored at −80°C for further experiments.

### 2.7 Immunofluorescence Analysis of Vero Cells Infected With Parental and Recombinant Virus

Vero cells were seeded in 12‐well plates and infected with either the parental and recombinant virus. At 72 h postinfection (pi), cells were fixed with 4% paraformaldehyde (GeneAll) for 15 min. Following fixation, cells were washed twice with phosphate‐buffered saline (PBS), permeabilized with 0.25% Triton X‐100 (BioShop, Canada) for 10 min, and blocked in PBS containing 2% bovine serum albumin (BSA; Geneall) for 1 h at room temperature (RT). Coverslips were incubated overnight at 4°C with anti‐flavivirus E monoclonal 4G2 antibody (Millipore, MAB10216‐I, MA, USA) diluted in PBS containing 2% BSA. After washing with PBS, the coverslips were incubated with Alexa fluor 488‐conjugated secondary antibody (Invitrogen, A11001, MA, USA) for 2 h at RT. Nuclei were stained with Hoechst 33258 (Thermo Fisher Scientific, MA, USA) for 15 min, washed with PBS and mounting onto microscope slides.

### 2.8 Plaque Assay for Quantification of JYJEV5 and Reporter Viruses

Plaque assays were performed to quantify parental and recombinant viruses, as detailed in the Supporting Information S1: Supporting Methods.

### 2.9 Cryo‐Electron Microscope (Cryo‐EM) of Parental and Recombinant Virus

The morphology of parental and recombinant viruses was examined by cryo‐electron microscopy, as detailed in the Supporting Information S1: Supporting Methods.

### 2.10 Determination of Lethal Dose (LD)_50_ and Neurovirulence Assessment in Mice

Eight‐week‐old female and male C57BL/6 mice (Samtako Bio, South Korea; *n* = 5 per group) were intracerebrally inoculated with serial doses of JYJEV3 (0.1, 1, 10, 10², and 10³ PFU) and JYJEV5 (1, 10, 10², 10³, and 10⁴ PFU), or with PBS only as a mock‐infected control. Virus stocks were prepared by serial dilution to the desired concentrations in PBS. Mice were monitored daily for 12 days to assess changes in body weight. Infection resulted in weight loss and disease manifestation, including hunched posture and ruffled fur. Mice exhibiting ≥ 20% body weight loss or signs of severe neurological impairment were euthanized using CO₂ asphyxiation, in accordance with guidelines approved by the Institutional Animal Care and Use Committee (IACUC) of Chungnam National University (Approval number: 202407A‐CNU‐116). The 50% LD_50_ was determined using the Finney's Probit Analysis method, and survival data were analyzed with GraphPad Prism version 8.0 (GraphPad Software, CA, USA).

### 2.11 Immunization and Challenge in Mice

C57BL/6 mice were used to evaluate the immunogenicity of the parental and recombinant viruses (JYJEV3 and JYJEV5). Virus stocks were diluted in PBS to the appropriate concentrations. The study included two PBS control groups, two JYJEV3 groups, two JYJEV5 groups, and one parental JEV5 group. The JYJEV3 and JYJEV5 groups were subdivided into two subgroups to assess cross‐protection, resulting in four experimental subgroups, while the JEV5 group served as a comparator for JYJEV5. Eight‐week‐old female C57BL/6 mice (*n* = 4 per group) were immunized via the intraperitoneal route with 1 × 10⁵ PFU of the respective viruses per dose at 2‐week intervals for a total of three doses. Control mice received equivalent volumes of PBS according to the same schedule and via the same route. The challenge experiment was conducted 1 week after the third immunization. Mice were intracerebrally challenged with JYJEV3 (250 PFU/20 µL) or JYJEV5 (2000 PFU/20 µL), representing 70 times the respective LD_50_ values. Mice were monitored daily for 12 days for weight loss and neurological symptoms, including tremors, ataxia, and paresis.

### 2.12 Enzyme‐Linked Immunosorbent Assay (ELISA) for Quantification of Virus‐Specific IgG in Immunized Mouse Sera

Virus‐specific IgG titers in immunized mouse sera were determined by ELISA, as detailed in the Supporting Information S1: Supporting Methods.

### 2.13 Serum Neutralization (SN) Assay Using Sera From Immunized Mice

SN assays were conducted to measure virus‐neutralizing antibody titers, as described in the Supporting Information S1: Supporting Methods.

### 2.14 Analysis of Viral Rna, Cytokine, and Chemokine Expression in Mouse Brain Tissue

Mouse brain tissues form JYJEV3 or JYJEV5‐infected and control mice were analyzed viral RNA, cytokine, and chemokine expression by quantitative RT‐PCR (qRT‐PCR) and Western blot analysis as described in the Supporting Information S1: Supporting Methods.

### 2.15 Determination of EC_50_ Values of NITD008 Against Recombinant Viruses

Vero cells were seeded in either 24‐well or black‐walled 96‐well plates and cultured to reach 80%–90% confluency before infection with recombinant viruses (JYJEV3, JYJEV5, JYJEV5‐NLuc, and JYJEV5‐EGFP) at a multiplicity of infection (MOI) of 0.1. After 1 h incubation at 37°C, the inoculum was removed, and the cells were washed twice with PBS. Cells were then treated with serial dilutions of NITD008 (MCE, HY‐12957, NJ, USA) in maintenance medium. At 72 h postinfection (hpi), virus replication was assessed according to the virus type. For JYJEV5‐NLuc, luciferase activity in the cell pellets was measured using the Nano‐Glo Luciferase Assay System (Promega) according to the manufacturer's protocol, and luminescence was detected using a VICTOR Nivo microplate reader (PerkinElmer, MA, USA). For JYJEV5‐EGFP, fluorescence intensity was directly measured in infected cells using the same reader. For wild‐type virus (JYJEV3 and JYJEV5), viral RNA was extracted from the supernatants using a commercial viral RNA extraction kit (Qiagen, Germany), followed by cDNA synthesis. Viral titers were determined by plaque assay, EC_50_ values were calculated using GraphPad Prism 8.0.

### 2.16 Statistical Analysis

Data are presented as mean ± standard deviation (SD). Statistical analyses were conducted using SPSS software (version 29; IBM, NY, USA). The normality of data distributions was assessed using the Shapiro–Wilk test. In vitro experiments were independently repeated at least three times. For comparison between two groups, an unpaired, two‐tailed Student's *t*‐test was used. For in vivo experiments, comparisons between groups (LD_50_, *n* = 5 per group; immunization, *n* = 4 per group) were performed using the Mann–Whitney *U* test for non‐parametric data, the unpaired two‐tailed Student's *t*‐test, Tukey's post hoc test, or the log‐rank (Mantel–Cox) test, as appropriate. *p* values less than 0.05 were considered statistically significant.

## 3 Results

### 3.1 Construction of the Full‐Length cDNA Clone of JEV5

Viral RNA was extracted from Vero cells infected with the genotype 5 JEV K15P38 strain and used to generate nine RT‐PCR fragments (A–I) that collectively span the entire genome of the virus. These individual fragments were subsequently cloned and assembled to construct the full‐length JEV cDNA of JEV5 (Supporting Information S1: Figure 1). A low‐copy‐number plasmid (pBAC) was selected for cloning. Three major JEV5 fragments were generated, namely fragment 1 (A + B + C), fragment 2 (D + E + F), and fragment 3 (G + H + I), each of which was inserted into pBAC vectors using the In‐Fusion cloning methods (Supporting Information S1: Figure 1). The final assembled construct contained a cytomegalovirus (CMV) promoter, a hepatitis deltavirus ribozyme (HDVr), and a bovine growth hormone (BGH) polyadenylation sequence at the 5′ and 3′ ends (Figure 1A). The successful assembly of the infectious clone was confirmed by agarose gel electrophoresis (Supporting Information S1: Figure 1C). The fully assembled clone was designated as pBAC‐JYJEV5. Sequence comparison between the assembled clone and the parental virus revealed two silent mutations, one missense mutation in the E gene, one missense mutation in the NS2B gene, and one silent mutation in each of the NS3 and NS4B genes, which were acquired unintentionally during the cloning process (Supporting Information S1: Table 3).

To evaluate the infectivity of the assembled clone, pBAC‐JYJEV5 was transfected into Vero cells. The transfected cultures were monitored daily for CPE, which was assessed in parallel with cells inoculated with the parent virus. CPE became visible in the infectious clone‐transfected cells starting on 5 days posttransfection, whereas the parent virus induced CPE beginning on day 4 postinfection. Viral protein expression in the transfected cells was analyzed by confocal microscopy using anti‐flavivirus E protein antibodies. Fluorescence signals corresponding to the viral E protein were observed from day 3 in both transfected and infected Vero cells (Figure 1C). These results confirm that transfection of pBAC‐JYJEV5 into Vero cells resulted in the generation of replication‐competent JEV5 particles, which exhibited CPE and viral protein expression comparable to those of the parent virus.

### 3.2 Comparison of Cell Culture Growth Between Parental and Recombinant JEV5

**Figure 2:**
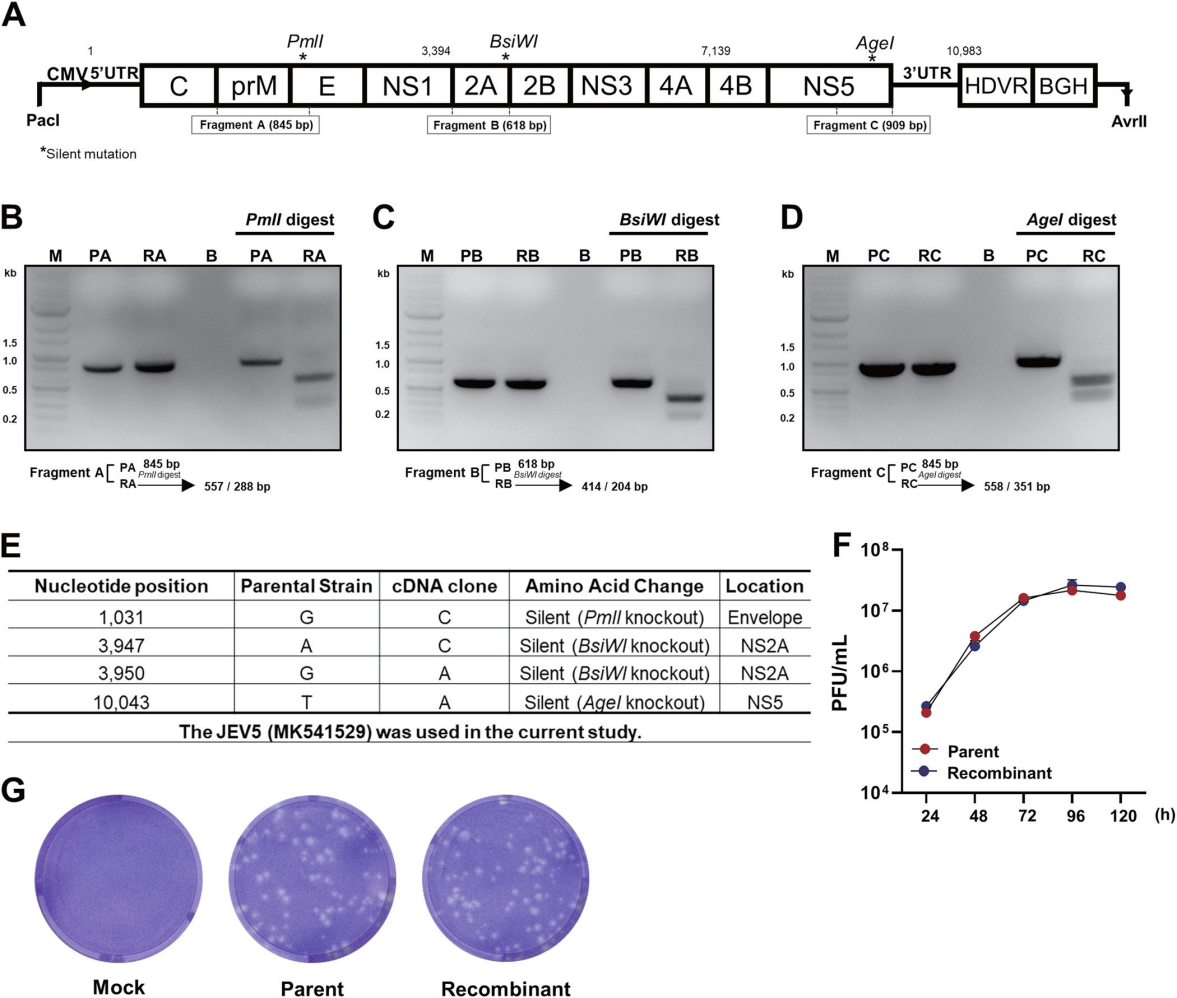
Molecular verification and in vitro characterization of recombinant JYJEV5. (A) Schematic representation of engineered silent mutations introducing restriction enzyme sites *PmlI* (E), *BsiWI* (NS2A), and *AgeI* (NS5) into the viral genome. (B–D) PCR amplification and subsequent restriction enzyme digestion confirming insertion of mutation sites in the recombinant virus. (E) Nucleotide sequence alignment displaying specific differences between the parental and recombinant viruses. (F) Multistep growth kinetics of parent JEV5 and recombinant JYJEV5 (MOI = 0.01) in Vero cells. Viral titers were measured by the TCID_50_ assay. (G) Plaque morphology of parental and recombinant viruses at 5 dpi. Parent, parent JEV5; Recombinant, JYJEV5; M, marker; B, empty well; PA, parent fragment A; RA, recombinant fragment A; PB, parent fragment B; RB, recombinant fragment B; PC, parent fragment C; RC, recombinant fragment C. Data represent at least three independent experiments.

To rule out the possibility of parental viral contamination, distinct point mutations were introduced into the E, NS2A, and NS5 genes via primer design to allow differentiation from the parental virus. Specifically, silent mutations were engineered to introduce a *PmlI* site (GACGTG → CACGTG), a *BsiWI* site mutation (AGTGCG → CGTACG), and an *AgeI* site mutation (TCCGGT → ACCGGT) (Figure 2A). To confirm the presence of these mutations, three RT‐PCR fragments spanning the specific regions were amplified from templates isolated from both parental and recombinant virus‐infected cells (Supporting Information S1: Table 2). The fragments included an 845‐bp fragment spanning nucleotides 477 to 1321 (fragment A), a 618‐bp fragment spanning nucleotides 3534 to 4151 (fragment B), and a 909‐bp fragment spanning nucleotides 9486 to 10 394 (fragment C). RT‐PCR products from the recombinant virus were digested with restriction enzymes to confirm the successful introduction of silent mutations. Fragment A (557 nt and 288 nt) was cleaved by *PmlI*, fragment B (414 nt and 204 nt) by *BsiWI*, and fragment C (558 nt and 351 nt) by *AgeI* (Figure 2B–E). These results confirmed the successful incorporation of the designated silent mutations into the engineered viral genome. Furthermore, full‐length sequencing of viral stocks at passages 20 confirmed that no additional mutations or reversions had occurred across the genome. In later passages of the reporter‐expressing virus, the expected deletion of the reporter gene cassette was also observed, consistent with reporter loss due to selective pressure. These results support the genetic stability and functionality of the engineered clone throughout serial passaging (Supporting Information S1: Data 1).

To evaluate viral replication, the growth kinetics of the parental and recombinant viruses were analyzed in Vero cells. As shown in Figure 2F, the parental virus exhibited accelerated replication dynamics during the initial 48 hpi, whereas the recombinant virus displayed a similar pattern but with a slight delay. However, both achieved comparable maximum titers at 72 hpi. Further analysis of viral titers from 72 to 120 hpi showed that both the parental and recombinant viruses reached comparable peak titers, reinforcing the genetic and phenotypic stability of the engineered clone. Plaque assays performed at 5 day‐post infection (dpi) showed no discernible differences in plaque size or morphology between the parental and recombinant viruses (Figure 2G). These findings indicate that the infectivity and replication kinetics of the recombinant virus closely resembled those of the parent viruses.

### 3.3 Morphological Comparison of Parent and Recombinant JEV5 Revealed by Cryo‐EM

**Figure 3:**
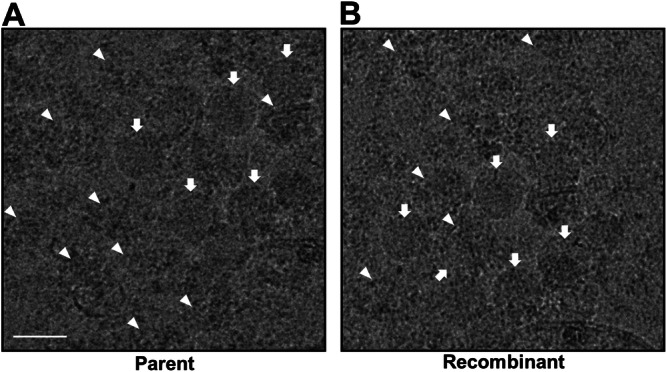
Morphological analysis of JYJEV5 by cryo‐EM. (A, B) Representative cryo‐EM images of parental JEV5 (A) and recombinant JYJEV5 (B) particles highlighting mature virions (white arrows) and immature virions (white triangles). Mature particles exhibit a smooth, spherical morphology with condensed internal electron density, indicative of fully processed envelope proteins. In contrast, immature particles display a rougher outer surface and less compact internal density, consistent with incomplete prM cleavage. Scale bar, 50 nm. Data represent at least three independent experiments.

To further characterize the recombinant virus, we analyzed the morphology of parental and recombinant viral particles using cryo‐EM. As shown in Figure 3, immature virions displayed uneven surface contours and were larger in diameter than their mature counterparts, which exhibited smooth, spherical surfaces (Figure 3A,B). The mean diameter of both virus types was approximately 50 nm. Cryo‐EM analysis confirmed the successful recovery of recombinant virus from the pBAC‐JYJEV5 construct and revealed structural features indistinguishable from those of the parental virus. These observations indicate that the recombinant virus faithfully mimics the parental virus in both immature and mature forms, with comparable ultrastructural properties.

### 3.4 Pathogenicity of JYJEV3 and JYJEV5 in Mice

**Figure 4:**
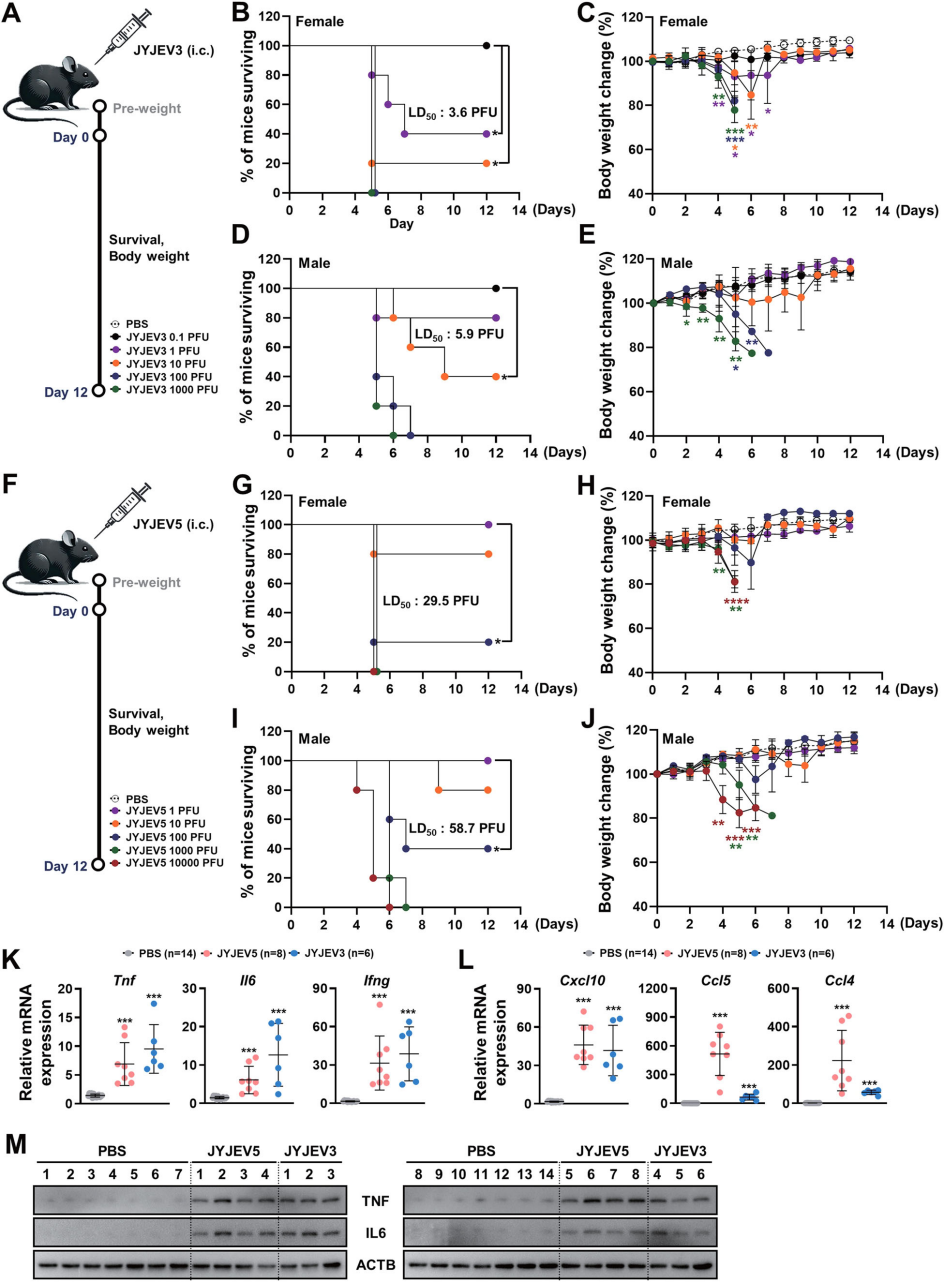
Neurovirulence assessment of JYJEV3 and JYJEV5 in mice. (A, F) Schematic overview of the intracerebral LD_50_ determination protocol in 8‐week‐old C57BL/6 mice. Group symbols and color codes are indicated. (B, D) Kaplan‐Meier survival curves for mice (*n* = 5 per group) inoculated intracerebrally with 10‐fold serial dilutions of JYJEV3 (B andD) or JYJEV5 (F and H), monitored for 12 days. (C and E) Corresponding changes in body weight postinoculation for JYJEV3 (C and E) and JYJEV5 (G and I). Statistical analysis was performed using the log‐rank test for survival data, and two‐way ANOVA followed by Tukey's post hoc test for body weight changes, comparing the PBS group with each infected group. *p < 0.05; **p < 0.01; ***p < 0.001; **** *p* < 0.0001. (K–M) Brain tissues were collected at the time of death from mice injected intracranially with PBS (*n* = 14), JEV5 (*n* = 8), or JEV3 (*n* = 6). (K and L) Total RNA was extracted, and mRNA expression levels of *Tnf*, *Il6*, *Ifng (K)*, *Cxcl10*, *Ccl5*, and *Ccl4* (L) were quantified using quantitative real‐time PCR. Expression levels were normalized to a housekeeping gene and are presented as relative expression compared to the PBS group. (M) Total protein was extracted, and TNF and IL6 expression was analyzed by Western blot. β‐actin was used as a loading control. Each lane represents an individual mouse. Data are presented as mean ± SD. Statistical analysis was performed using one‐way ANOVA followed by Tukey's post hoc test to compare PBS with each infected group (PBS vs. JYJEV5, PBS vs. JYJEV3). ***p < 0.001.

Previously, we developed a JEV3 recombinant virus, JYJEV3, which was evaluated for vaccine potential [33]. In the present study, we employed the same reverse genetics system to generate JYJEV5 and assess the efficacy of this clone against various genotypes. To evaluate the infectious potency of JYJEV3 and the newly constructed JYJEV5, we measured the LD_50_ in 8‐week‐old C57BL/6 female and male mice. Groups of mice (*n* = 5) were inoculated intracerebrally with decreasing doses of JYJEV3 (10^3^, 10^2^, 10^1^, 1, and 0.1 PFU) or JYJEV5 (10^4^, 10^3^, 10^2^, 10^1^, and 1 PFU) (Figure 4A). Body weight was monitored daily for 12 days postinoculation to assess survival.

All mice in the JYJEV3 groups inoculated with 10³ and 10² PFU exhibited significant weight loss and disease symptoms, such as ataxia or tremors, and were euthanized on day 5 to 7 postinfection due to > 20% weight loss (Figure 4B–E). In the 10^1^ PFU group, four out of five female mice died, resulting in a survival rate of 20%, whereas three out of five male mice died, resulting in a survival rate of 40%. In the 1 PFU group, one female mouse died on each of days 5, 6, and 7, leaving only two survivors for a 40% survival rate, whereas in males only one mouse died on Day 5, resulting in an 80% survival rate. All the mice in the 0.1 PFU group survived, demonstrating a 100% survival rate in both females and males (Figure 4B–E).

Similarly, for JYJEV5, all female and male mice exposed to 10^4^ and 10^3^ PFU died within the same observation period. In the 10² PFU group, three of five female mice died by Day 5 and one more died on Day 7, resulting in a 20% survival rate, whereas two of five male mice died on Day 6 and one more died on Day 7, yielding a 40% survival rate. In the 10^1^ PFU group, one of five female mice died on Day 5 and one of five male mice died on Day 9, yielding an 80% survival rate in both groups. All female and male mice in the 1 PFU group survived throughout the observation period (Figure 4F–I). Based on the results, the calculated LD₅₀ for JYJEV3 was approximately 3.6 PFU in females and 5.9 PFU in males, whereas the LD₅₀ for JYJEV5 was approximately 29.5 PFU in females and 58.7 PFU in males. In parallel, body temperature monitoring revealed transient fever responses preceding weight loss in virus‐inoculated mice, followed by a gradual decline in temperature, indicative of impaired thermoregulatory function as encephalitic symptoms progressed (Supporting Information S1: Figure 2). These findings indicate that JYJEV3 exhibits greater virulence than JYJEV5 in both female and male mice, as assessed by intracerebral inoculation. Cytokines and chemokines play important roles in JEV‐induced neuropathology, including glial activation, blood–brain barrier disruption, and recruitment of inflammatory cells to the CNS [34]. To investigate genotype‐specific immune responses, we measured mRNA expression of cytokines (*Tnf*, *Il6*, and *Ifng*) and chemokines (*Cxcl10*, *Ccl5*, and *Ccl4*) in brain tissues from mice infected with JEV5 or JEV3 by quantitative RT‐PCR (Figure 4K,L). Both JEV5 and JEV3 infections significantly upregulated *Tnf*, *Il6*, and *Ifng* expression compared to uninfected controls (Figure 4K). *Cxcl10* expression was also significantly increased in both JEV5‐ and JEV3‐infected mice relative to controls (Figure 4L). Notably, *Ccl5* and *Ccl4* mRNA levels were significantly elevated in JEV5‐infected mice compared to controls, with a trend toward higher expression than in JEV3‐infected mice (Figure 4L). To further validate cytokine induction at the protein level, we examined TNF and IL‐6 expression in brain tissues by Western blot analysis (Figure 4M). Consistent with the mRNA data, both cytokines were upregulated in JEV5‐ and JEV3‐infected mice compared to the control mice. These results indicate that both genotypes induce robust pro‐inflammatory cytokine responses, while JEV5 infection may preferentially enhance chemokine expression related to immune cell recruitment.

### 3.5 Immunization and Challenge Study in Mice

**Figure 5:**
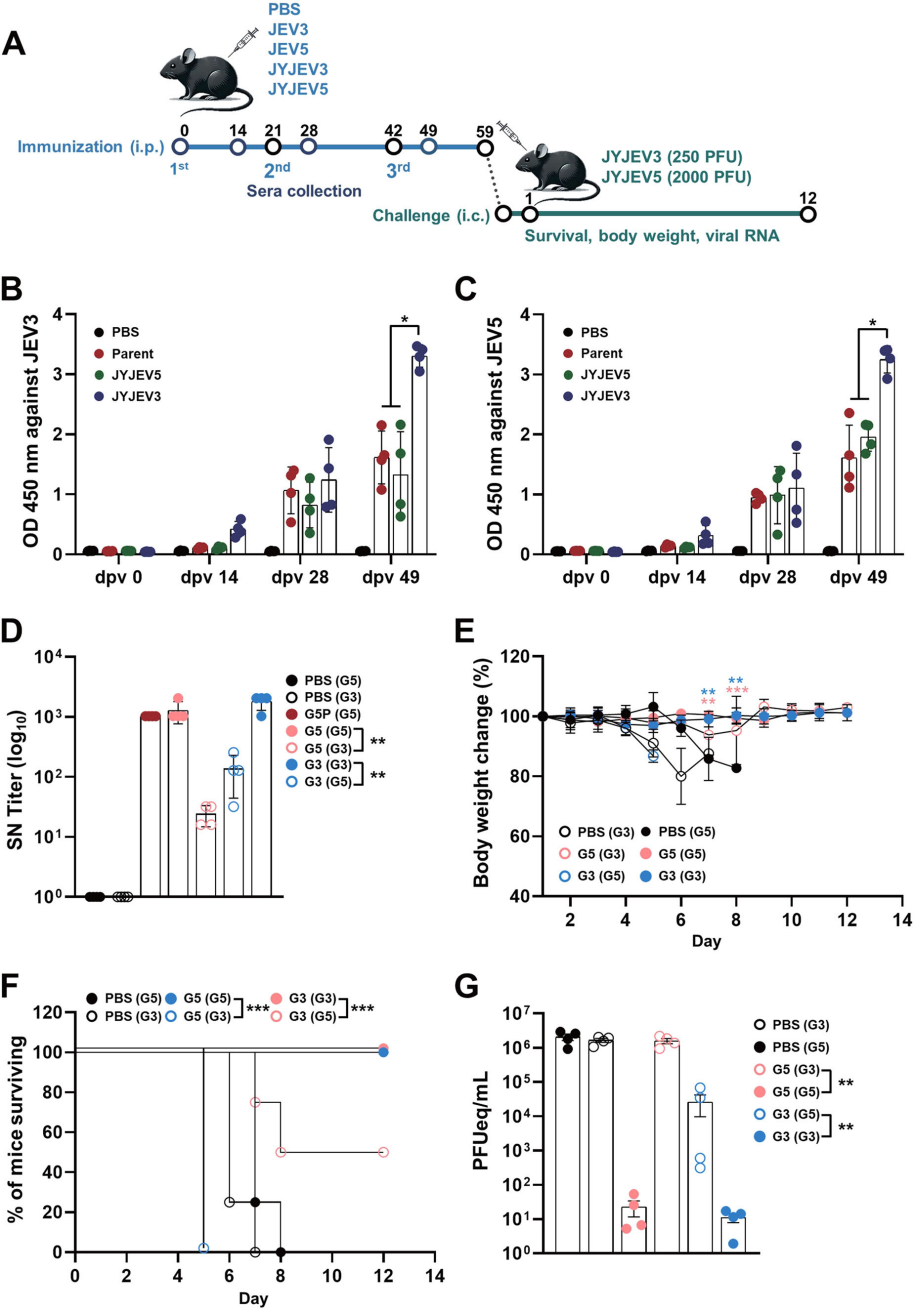
Immunogenicity and protective efficacy of JYJEV5 in mice. (A) Schematic of the immunization and challenge schedule. Five‐week‐old C57BL/6 mice (*n* = 4 per group) were immunized intraperitoneally (5 × 10^5^ PFU) with PBS (negative control), parental virus (JEV5), or recombinant viruses (JYJEV3 and JYJEV5). Serum samples were collected on days 0, 21, 28, 35, 42, and 49 post‐immunization. (B and C) JEV3 and JEV5‐specific IgG titers were quantified by ELISA. (D) Neutralizing antibody titers (NT_50_) were determined using a serum neutralization assay. Data are shown as mean ± SD (*n* = 4). (E and F) On day 49 post‐immunization, mice were challenged intracerebrally with either JEV3 (250 PFU) or JEV5 (2000 PFU). (E and F) Body weight (E) and survival rates (F) were monitored for 12 days post‐challenge (Kaplan–Meier plot). Statistical analysis was performed using the two‐way ANOVA followed by Tukey's post hoc test for body weight changes, comparing the homologous with heterologous groups. (G) At the endpoint, mice were euthanized, and brain tissues were collected for viral RNA quantification by qRT‐PCR. Viral RNA levels were expressed as PFU equivalents per mL (PFUeq/mL), based on standard curves generated from PFU‐titrated virus stocks. Group labels are shown as “X (Y)”, where X indicates the immunization group and Y denotes the challenge virus genotype. Statistical analysis was performed using the Mann–Whitney U test or the log‐rank test, as appropriate. *p < 0.05; **p < 0.01; ***p < 0.001. G3, JEV3; G5, JEV5; ip, intraperitoneally; ic, intracerebrally.

To assess the vaccine potential of viruses derived from infectious clones, C57BL/6 mice (*n* = 4) were immunized intraperitoneally with 1 × 10⁵ PFU of each virus at 2‐week intervals for a total of three doses, and subsequently evaluated for immune responses. The immunization groups included PBS (negative control), parent virus, JYJEV5, and JYJEV3. Serum samples were collected on days 0, 14, 29, and 49 postvaccination (dpv) to evaluate IgG titers. In addition, SN test was performed at 49 dpv to assess the functional neutralizing antibody responses. Following the third immunization, mice were challenged either homologously or heterologously depending on the group, and monitored daily for survival and body temperature monitoring. Further details of the immunization and challenge protocols are provided in Figure 5A.

Analysis showed an initial antibody response against JYJEV3 and JYJEV5, detectable from the first immunization. As the immunization schedule progressed, IgG titers increased consistently, indicating a robust and cumulative immune response. Although cross‐reactivity was observed between JYJEV3 and JYJEV5, JYJEV3‐immunized group at 49 dpv exhibited particularly robust IgG responses, suggesting that JYJEV3 immunization elicited a stronger humoral response (Figure 5B,C, and Supporting Information S1: Figure 3).

Serum neutralization assays were conducted using 49‐dpv serum samples to evaluate immune efficacy further. The parent JEV5 and JYJEV5 groups exhibited neutralization titers ranging from 1024 to 2048 against JYJEV5. Similarly, the JYJEV3 group showed neutralization titers of 1024–2048 against JYJEV3. In contrast, JYJEV3‐immune sera neutralized JYJEV5 at reduced titers of 32–128, while JYJEV5‐immune sera showed even lower neutralization titers of 16–32 against JYJEV3, highlighting the limited cross‐protection immunity and antigenic mismatch between genotypes (Figure 5D). In the intracerebral challenge experiments, all mice in the negative control group exhibited progressive weight loss and succumbed to infection with JYJEV3 and JYJEV5 infections by Days 5 and 6, respectively. In contrast, all mice immunized with the recombinant JYJEV5 survived homologous challenge with JYJEV5, and those immunized with JYJEV3 also survived homologous challenge with JYJEV3, indicating robust protective immunity. However, all JYJEV5‐immunized mice exhibited body weight loss and succumbed to JYJEV3 challenge by Day 5, whereas two of four JYJEV3‐immunized mice also showed body weight loss and succumbed to heterologous JYJEV5 challenge, with the remaining two surviving (Figure 5E,F and Table 1).

**Table 1:** Survival reates of mice immunized with JEV3 or JEV5 following homologous or heterologous challenge.

Group		Treatment → Challenged	Number		Survival rate (%)
			Total	Survival	
PBS	G3	Control (PBS) → G5 (2000 PFU)	4	0	0
	G5	Control (PBS) → G3 (250 PFU)	4	0	0
G5	G5	G5 → G5 (2000 PFU)	4	4	100
	G5	G5 → G3 (250 PFU)	4	0	0
G3	G3	G3 → G5 (2000 PFU)	4	2	50
	G3	G3 → G3 (250 PFU)	4	4	100

Viral titers in brain samples from challenged mice were assessed by real‐time PCR. As shown in Figure 5G, mice in the control group exhibited high viral titers following challenge with either JYJEV3 or JYJEV5, indicating a lack of protective immunity. In contrast, mice immunized with either JYJEV3 or JYJEV5 and challenged with the homologous strain showed markedly reduced viral titers in the brain, demonstrating effective protection. However, mice immunized with JYJEV3 or JYJEV5 and challenged with the heterologous strain exhibited elevated viral titers, comparable to those observed in non‐immunized controls, indicating limited cross‐genotype protective immunity between JYJEV3 and JYJEV5. These results confirmed restricted cross‐protection between JYJEV3 and JYJEV5, as reflected by both neutralization titers and survival rates.

### 3.6 Construction and Characterization of JYJEV5 Infectious Clones With Reporter Genes

**Figure 6:**
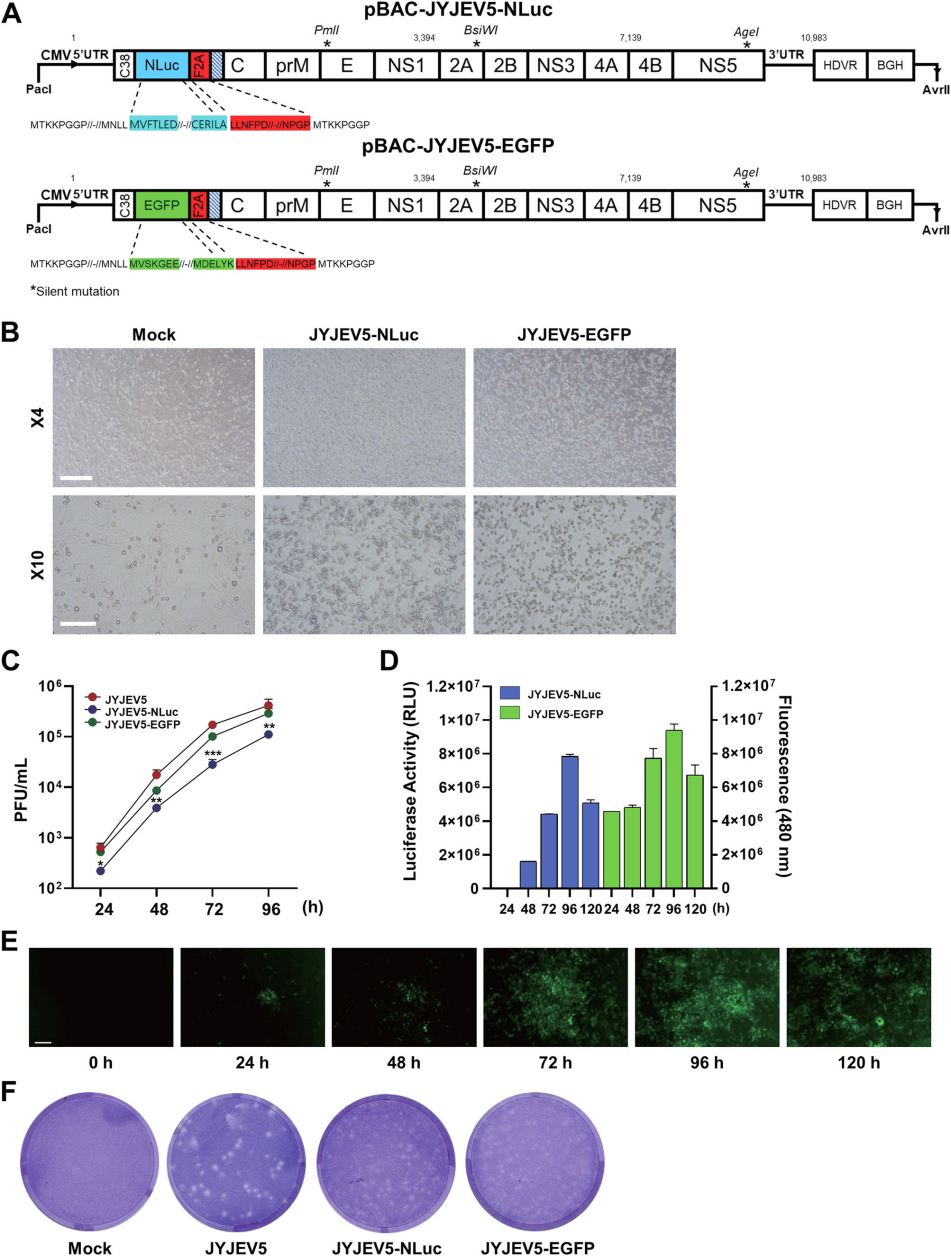
Characterization of EGFP‐ and NLuc‐expressing JYJEV5 reporter viruses. (A) Schematic diagram of the reporter gene insertion strategy. EGFP or NLuc, along with C38 and FMDV‐2A self‐cleaving peptide, was inserted between the 5′ UTR and the capsid gene. (B) CPE effects in Vero cells at 5 dpt with recombinant plasmids, shown at ×4 (upper panels) and ×10 (lower panels) magnifications. Scale bar, 200 μm (×4), and 400 μm (×10). (C) Growth kinetics of reporter viruses in Vero cells. Cells were infected at an MOI of 0.1, and viral titers were determined at each time point. (D) Quantification of reporter signal from 24 to 120 hpi using a plate reader. (E) Time‐lapse microscopy of EGFP expression in infected Vero cells. Scale bar, 400 μm. (F) Plaque morphology at 5 dpi. Data represent means ± SD from three replicates. *p < 0.05; ** *p* < 0.01; ***p < 0.001.

To validate the functionality of our reverse genetics system, we utilized the infectious clone pBAC‐JYJEV5 to generate cDNA clones expressing the EGFP and NLuc reporter genes. As shown in Supporting Information S1: Figure 4, three fragments were inserted between the 5′ UTR and the capsid gene, including the N‐terminal 38 amino acids of the capsid protein (C38), the EGFP or NLuc gene, and the FMDV‐2A sequence (Figure 6A). The C38 region contains essential cis‐acting RNA elements required for genome cyclization [35]. To ensure efficient processing, the FMDV‐2A cleavage site was incorporated [36]. The downstream C38 coding sequence was synonymously altered to prevent homologous recombination while preserving the amino acid sequence. Recombinant reporter viruses were transfected into Vero cells, and CPE was continuously monitored. By day 5 posttransfection, pronounced CPE was evident, confirming successful viral replication (Figure 6B).

To evaluate the genetic stability of the inserted reporter gene, the rescued virus (P0) was blindly passaged in Vero cells for 10 consecutive generations (P1 to P10) (Supporting Information: Figures 5A, 6A). Viral RNA was extracted from the culture supernatant of each passage, and RT‐PCR was performed using primers designed to amplify the region spanning from the 5′UTR to the C gene of the JEV5 genome. The expected products for JYJEV5‐NLuc and JYJEV5‐EGFP were about 1100 and 1304 bp, respectively. In the case of the NLuc‐expressing virus, a distinct 513 bp, which corresponds to the viral genome lacking the NLuc insert, first appeared at P6, while the intensity of the full‐length 1100 bp amplicon gradually diminished from P6 to P10 (Supporting Information S1: Figure 5B, Supporting Information S1: Data 2). This pattern indicates that the inserted reporter gene exhibited decreasing genetic stability over successive passages, likely due to selective pressure against the additional sequence, resulting in progressive excision from the viral genome. Consistent with the RT‐PCR findings, NLuc luminescence activity remained stable until P5 but declined markedly from P6 onward, confirming a loss of functional reporter gene expression over time. Similarly, in the EGFP‐expressing virus, an additional RT‐PCR band of approximately 695 bp—indicative of reporter gene deletion—was detected as early as passage (P) 1, with its intensity gradually increasing over subsequent passages up to P10 (Supporting Information: Figure 6B, Supporting Data 3). The EGFP gene was consistently detected by PCR from P1, but the intensity of the EGFP‐containing band decreased while the deletion band intensified through P10, indicating progressive loss of the reporter gene. Despite this, fluorescence signals remained stable, suggesting that JYJEV5‐EGFP exhibited a slower decline in stability compared to NLuc. Based on these observations, P4 was selected as the representative virus stock for subsequent experiments, as it retained detectable reporter gene expression while minimizing the influence of genetic instability.

**Figure 7:**
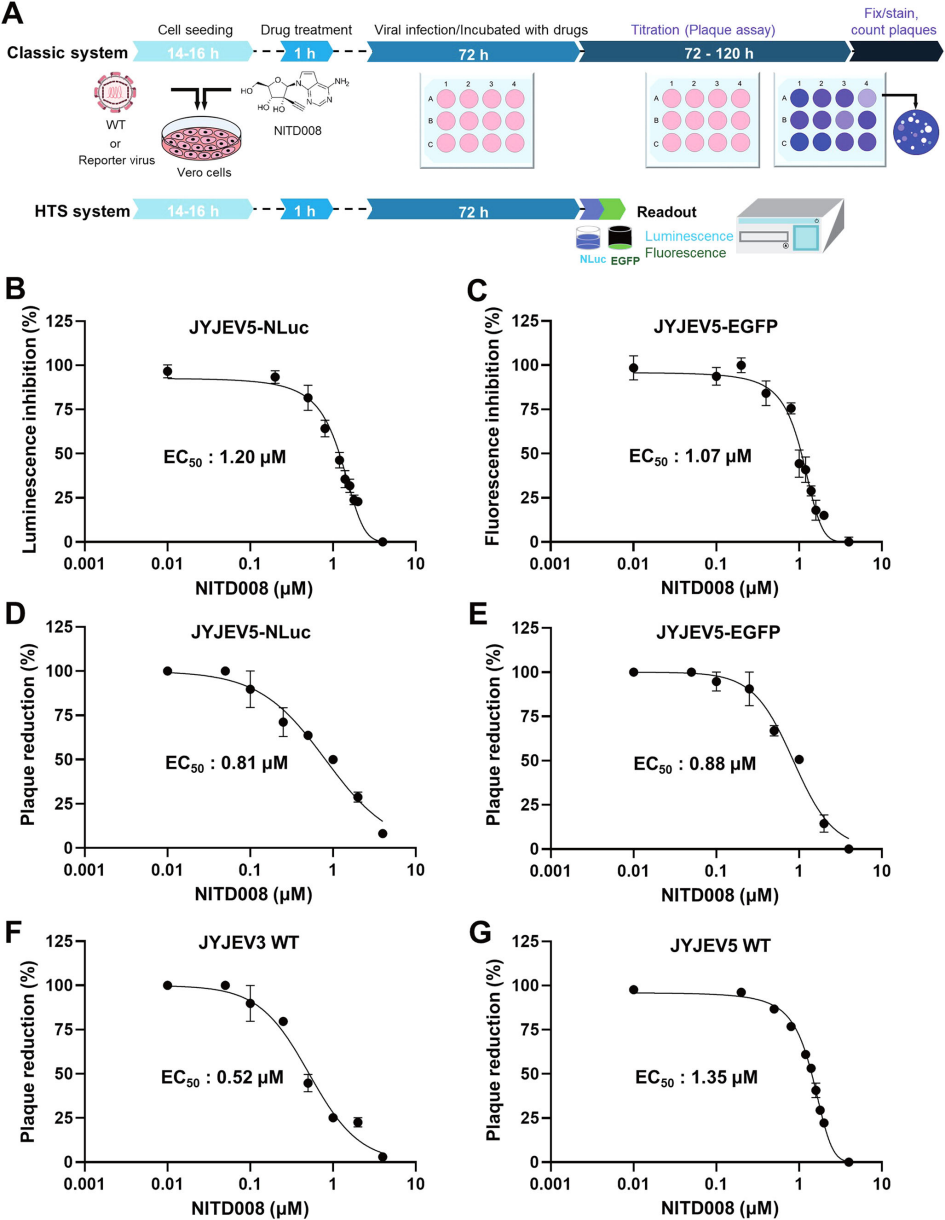
Evaluation of antiviral efficacy using a reporter virus‐based JEV5 assay and comparison with classical plaque assay. (A) Schematic overview of two antiviral screening workflows. In the classical method (upper), the JYJEV5 reporter virus (MOI = 0.1) was incubated with serially diluted NITD0008 for 1 h at 37°C. The mixture was then used to infect Vero cells. At 72 hpi, supernatants were collected, and viral titers were quantified by plaque assay. In the reporter‐based HTS system (lower), NLuc activity or EGFP fluorescence was directly measured from infected cells at 72 hpi using a plate reader. (B and C) Dose‐dependent antiviral effect of NITD008 in cells infected with recombinant virus (JYJEV5‐NLuc and JYJEV5‐EGFP) at an MOI of 0.1. EC_50_ values were determined based on reduction in NLuc activity (B) and fluorescence (C), relative to DMSO‐treated controls. (D and E) Dose‐dependent antiviral effect of NITD008 against JYJEV5‐NLuc (D) and JYJEV5‐EGFP (E), determined by plaque reduction assay in Vero cells infected at an MOI of 0.1, relative to DMSO‐treated controls. (F and G) EC_50_ values of NITD008 were determined for JYJEV3 (F) and JYJEV5 (G) using plaque reduction assay. All data are presented as mean values from at least three independent experiments.

Growth kinetics were quantified by measuring viral titers from 24 to 96 hpi. Although titers of both reporter viruses increased over time, their peak titers were lower than those of the wild‐type recombinant viruses. The NLuc‐containing clone exhibited reduced viral titers, suggesting that NLuc insertion more significantly attenuated replication than EGFP (Figure 6C). NLuc activity and EGFP fluorescence increased in a time‐dependent manner from 24 to 96 h, then declined at 120 h (Figure 6D). To further support the reliability of the reporter system for high‐throughput screening applications, we performed linear regression analysis comparing luciferase activity and EGFP fluorescence with corresponding viral titers. Strong correlations were observed, with *R*² values of 0.9054 for NLuc and 0.9035 for EGFP, respectively (Supporting Information S1: Figure 7A,B). EGFP fluorescence was also visualized by the microscope, yielding results consistent with those obtained by the plate reader (Figure 6E). These findings confirmed that both reporter viruses stably expressed their respective reporter genes.

Plaque assays revealed differences in plaque morphology. In Figure 6F, the wild‐type virus produced well‐defined circular plaques, whereas reporter gene‐containing viruses generated smaller, less‐distinct plaques with unclear boundaries. These findings further support the attenuating effect of reporter gene insertion.

### 3.7 Application of the JYJEV5 Reporter Virus System for Antiviral Drug Discovery

To assess the utility of the JYJEV5 reporter virus system for antiviral compound screening, we evaluated the efficacy of NITD008, a pan‐flavivirus nucleoside analog inhibitor [37]. As shown in Figure 7A, we compared two experimental workflows. One was the classical plaque assay, and the other was the reporter‐based HTS system. In the classical assay, Vero cells were infected with the virus and treated with NITD008. After incubation, supernatants were harvested, and viral titers were then determined by a plaque assay, which is a labor‐intensive process. By contrast, the HTS system employed JEV5 strains expressing NLuc or EGFP, enabling direct quantification of antiviral activity via luminescence or fluorescence signals, thus significantly reducing experiment time and complexity.

Using the reporter system, we directly assessed the antiviral efficacy of NITD008 and compared the results with those from the plaque assay. Treatment of infected Vero cells resulted in dose‐dependent suppression of JYJEV5‐NLuc or JYJEV5‐EGFP signals, indicating effective inhibition. The NLuc‐based assay yielded an EC_50_ of 1.20 μM (Figure 7B), and the EGFP‐based assay showed an EC_50_ of 1.07 μM (Figure 7C). To validate these findings, plaque inhibition assays with the same reporter viruses yielded EC_50_ values of 0.81 μM (Figure 7D) and 0.88 μM (Figure 7E), respectively. Additionally, plaque reduction assays with NITD008 against JYJEV3 and JYJEV5 showed EC_50_ values of 0.52 μM (Figure 7F) and 1.32 μM (Figure 7G), indicating greater sensitivity of JYJEV3. Importantly, EC_50_ values obtained from the reporter virus assays were closely aligned with those from classical plaque reduction assays, confirming the accuracy and applicability of the reporter system for antiviral evaluation. Collectively, the results show that NLuc‐ or EGFP‐expressing JEV5 provides a robust and efficient system for antiviral compound evaluation.

## 4 Discussion

Currently, JE vaccines are the only available methods for preventing JEV infection, and no antiviral therapeutics are available yet [27, 38]. While existing vaccines have shown high effectiveness against historically prevalent JEV genotypes, such as JEV1 and JEV3, the emergence of the JEV5 has raised concerns regarding vaccine efficacy [39]. Several studies have shown that JEV3‐based vaccines offer sufficient cross‐reactivity against JEV1 to JEV4 [25, 40]. However, the currently available JE vaccines do not provide adequate protection against the emerging JEV5 [41]. The key reason for this limited protection lies in the genetic divergence among JEV genotypes. The nucleotide sequence identity of the E gene between the Muar strain (JEV5) and JEV1 to JEV4 ranges from 78.6% to 79.7%, and the amino acid identity ranges from 90.0% to 91.6%, which indicates substantial genetic divergence between JEV5 and other established genotypes [42]. Since the JEV E protein is essential for eliciting neutralizing antibodies, the remarkable sequence divergence observed in the JEV5 strains results in limited cross‐reactivity with antibodies induced by other genotypes [43]. Previous studies have highlighted that even minor genetic variations in flaviviruses can significantly alter their antigenicity and immune recognition [44, 45]. The diminished neutralization capacity could reduce vaccine effectiveness and potentially increase the risk of infection in vaccinated populations, especially in regions where JEV5 circulates [25]. Considering the rapid evolution of JEV and the potential for new genotypes to emerge, a reassessment of the antigenic composition of current vaccines is crucial.

The yeast artificial chromosomes (YAC) and BAC systems enable the stable cloning and modification of large DNA fragments, making them valuable tools for generating full‐length viral cDNA clones [46]. However, YAC systems often require more complex handling and yeast‐specific tools, and are frequently less stable during propagation. In contrast, BACs allow robust propagation in *E. coli* and offer a straightforward workflow [46]. These advantages have made them a widely adopted platform for constructing infectious clones of various viruses, including JEV, as demonstrated in previous studies [47]. In the present study, we developed a reverse genetics system for JEV5 isolates from South Korea. Although an infectious clone of JEV5 was previously developed using the Muar strain isolated in Malaysia in 1952 [48], our system is based on contemporary JEV5 strains currently circulating in South Korea, representing a more recent and regionally relevant lineage [49]. Considering the potential genetic and phenotypic differences between historical and modern JEV5 strains, our platform provides a timely and applicable molecular tool for current studies on JEV5 pathogenesis, vaccine development, and antiviral screening. The system enabled the generation of synthetic viruses from genetically stable full‐length infectious JEV5 cDNAs. A CMV promoter sequence was added to the 5′ end of JYJEV5 genomic cDNA, and an HDVr sequence was added to the 3′ end to generate a natural 3′ terminus of the virus. The recombinant virus demonstrated infectivity in Vero cells comparable to that of the parent virus, as confirmed by similar patterns of CPE formation and viral protein expression. In addition, we differentiated the recombinant virus from the parent strain by introducing specific point mutations in the E, NS2A, and NS5 genes. This strategy not only enabled us to confirm the identity of the recombinant virus and exclude contamination, but also to establish a platform for flexible modification of the infectious clone. This platform facilitates future manipulations, including site‐directed mutagenesis, reporter gene insertion, and the generation of chimeric viruses for functional or immunological studies. Successful incorporation of these silent mutations was confirmed by restriction enzyme digestion, which yielded distinct fragment patterns at each targeted site. We closely monitored the growth kinetics of both parental and recombinant JEV5 (JYJEV5) in Vero cells. A comparison of replication curves, plaque morphology, and cryo‐EM results revealed no significant difference between the two. These results suggest that JYJEV5 retains key biological features of the parental virus, supporting its utility as a surrogate model for further investigation and preliminary vaccine evaluation.

To evaluate genotype‐specific differences in pathogenicity, we compared the neurovirulence of JYJEV5 and JYJEV3 in a murine model. Previous studies have demonstrated that the genetic background of the host is an important determinant of susceptibility to viruses, with different mouse strains showing distinct outcomes upon infection [50]. Specifically, C3H/He mice suffered from both high infection rates and mortality. C57BL/6, RR, NC, and KK mice showed similar infection rates to C3H/He but significantly lower mortality. In contrast, AA, BALB/c, and ddY mice exhibited the lowest infection rates and no mortality [51]. In light of these observations, we selected C57BL/6 as a well‐characterized model with intermediate susceptibility for our study. Moreover, C57BL/6 mice are widely used in JEV pathogenesis research across numerous studies [52], facilitating direct comparisons with existing literature. Based on intracerebral infection results in both female and male mice, JYJEV3 exhibited significantly higher virulence than JYJEV5, as indicated by a lower LD₅₀ value. These findings were considered in light of previous studies examining genotype‐specific differences in JEV pathogenicity using various experimental approaches. Differences in virulence between JEV5 and JEV3 are likely related to genotype‐specific infection characteristics. These differences may arise from variations in cellular tropism, neuro‐invasiveness, and neurovirulence, all of which are influenced by viral genetics and host interactions. For instance, JEV3 has shown higher infectivity in brain microvascular endothelial cells in vitro, suggesting enhanced brain blood‐barrier penetration. In contrast, some JEV5 displayed higher virulence than JEV3 following intraperitoneal inoculation in mice [53]. Moreover, virulence can vary even within the JEV5, indicating that intra‐genotypic differences may also influence pathogenic potential [43].

Our immunization study demonstrated that both JYJEV3 and JYJEV5 elicited significant immunogenicity, as shown by a progressive increase in IgG titers after serial immunizations. Parental and recombinant viruses induced high neutralization antibody titers against homologous viruses, confirming their potential as effective vaccines. However, challenge studies revealed limited cross‐protection between the JEV5 and JEV3. Although immunization conferred protection against homologous challenges, protection against heterologous genotypes was substantially lower. These findings underscore genotype‐specific immune responses observed in this model and suggest that developing vaccine strategies capable of broader genotype coverage may be warranted.

Our findings are consistent with previous studies that reported limited cross‐reactivity among JEV genotypes. JEV3‐based vaccines have been shown to exhibit reduced efficacy against JEV5, reinforcing the need for genotype‐matched vaccines [41, 54]. Notably, the re‐emergence of JEV5 in regions such as China and South Korea after decades of absence underscores the urgency for updated surveillance and vaccine policies. As JEV5 continues to spread and co‐circulate with other genotypes, the long‐term effectiveness of current monovalent vaccines may become increasingly limited [55]. Furthermore, the emergence of JEV4 and JEV5 introduces new challenges for JE control, calling for updates to vaccine formulations [23, 56, 57, 58]. To address this challenge, updated vaccines incorporating JEV5 antigens, or multivalent formulations targeting multiple circulating genotypes, should be considered to enhance protective breadth [59]. Alternatively, next‐generation vaccine platforms that target conserved epitopes across genotypes may offer broader and more durable protection, especially in endemic areas with increasing genotype diversity [60]. However, the effectiveness of such genotype‐inclusive strategies may be shaped by pre‐existing immunity, especially in individuals previously exposed to heterologous JEV strains [24]. Similar to observations in dengue virus, where pre‐existing immunity to one serotype can influence responses to heterologous serotypes [61], individuals with prior exposure to JEV1 or JEV3 may exhibit altered immune responses to JEV5‐based vaccines. Such effects could manifest through immune imprinting or antibody interference, potentially affecting both the magnitude and quality of vaccine‐induced immunity [62]. Although independent replicate in vivo experiments were not conducted, the consistent findings within each group (*n* = 4 or 5) support the robustness of the observed immunological trends and reinforce the validity of our conclusions. In light of these findings, future vaccine strategies should take into account the impact of pre‐existing immunity, particularly in regions where multiple JEV genotypes co‐circulate.

Traditionally, antiviral drug screening involves a series of in vitro assays to evaluate the ability of a compound to inhibit viral replication [63]. Among these, the plaque reduction assay is widely used to quantify antiviral activity by measuring reductions in plaque formation [64]. Although this assay is well‐established and reliable, it is labor‐intensive and time‐consuming. It requires compound‐virus incubation, infection, and a long period of incubation for plaque development before quantification [65, 66]. To overcome these limitations, reporter gene‐based assays have been developed as efficient alternatives. These assays measure viral replication through fluorescent or luminescent signals, providing rapid readouts and reducing assay time [32, 66].

Building upon this system, we next incorporated reporter genes into the JYJEV5 clone. The resulting reporter viruses expressed either NLuc or EGFP and enabled real‐time visualization and quantification of viral replication. Previous studies have reported that placing reporter genes at the 5′ UTR–capsid junction results in greater stability, particularly when sequence homology between duplicated capsid regions is minimized [35]. Nevertheless, reporter gene insertion has also been shown to influence viral fitness, such as reduced replication, smaller plaque size, or altered cytopathic effects, likely due to disruptions in genome cyclization or translation kinetics. In our study, both EGFP‐ and NLuc‐expressing viruses exhibited modest attenuation in viral titers and plaque size compared to the parental clone, with the NLuc‐containing virus showing a more pronounced reduction in replication efficiency. Furthermore, we observed genetic instability of the reporter gene over multiple passages, with partial or complete loss of the inserted gene likely caused by selective pressure against foreign sequences. Such instability has also been reported in other flavivirus reporter systems [67], and several approaches have been proposed to mitigate this, including the use of small peptide reporters to minimize genomic instability [68], and extending the duplicated capsid region to enhance stability and suppress recombination‐mediated loss [35]. Despite these efforts, reporter gene–bearing viruses often remain attenuated compared to their wild‐type counterparts, which may complicate studies on viral replication, pathogenesis, or transmission [67]. Therefore, recognizing the inherent limitations of current reporter virus systems is essential, and continued refinement of vector design is necessary to improve their stability and biological relevance. Our results demonstrate that reporter virus systems are reliable and efficient alternatives to classical plaque assays. Strong correlations were observed between luminescence‐ or fluorescence‐based assays and plaque assays. These findings support the use of JYJEV5‐NLuc and ‐EGFP expressing strains as valuable tools for high‐throughput antiviral screening and flavivirus drug discovery.

In conclusion, we successfully generated a full‐length infectious clone of isolated JEV5. This system advances our understanding of the molecular and pathogenic properties of emerging JEV genotypes and provides a critical platform for studying viral replication, immune escape, and virulence. Furthermore, the clone can be applied to evaluate vaccine efficacy and antiviral strategies. Although the present study focused on JEV3 and JEV5, future work incorporating JEV1 and other circulating genotypes will be essential to more comprehensively assess cross‐genotype protection and broaden vaccine coverage. Future studies may employ this system to investigate the impact of specific genetic variations on viral fitness and host responses. These insights may contribute the development of next‐generation vaccines and targeted therapies.

## Author Contributions

J.‐Y.P. conceived and designed experiments. H.‐M.L. and H.‐J.S. project administration. J.‐Y.P., W.K., and H.‐M.L. performed the experiments. J.‐Y.P. and H.‐M.L. analyzed the data. J.‐Y.P. and H.‐M.L. wrote the paper. H.‐M.L. and H.‐J.S. acquire funding. All the authors reviewed and approved the final version of the manuscript.

## Conflicts of Interest

The authors declare no conflicts of interest.

## Supporting information


**Supporting Information Figure 1.** Construction of the pBAC‐JYJEV5 infectious clone. (A) Schematic representation of the pBAC‐JYJEV5 construct comprising three fragments. (B) PCR amplification of each fragment was resolved on an agarose gel. (C) Agarose gel electrophoresis confirmed PCR amplification of each fragment. M, marker; N, non‐template control; F1, Fragment 1; F2, Fragment 2; F3, fragment 3. **Supporting Information Figure 2.** Physiological responses following intracerebral inoculation with JYJEV3 and JYJEV5. (A) Schematic representation of experimental design. Male and Female C57BL/6 mice (*n* = 5 per group) were intracerebrally injected with PBS, JYJEV3 (250 PFU), or JYJEV5 (2000 PFU) at a dose corresponding to 70‐fold of the LD_50_ and monitored for 12 days. (B and C) Changes in body weight (B) and body temperature (C) in female mice. (D and E) Changes in body weight (D) and body temperature (E) in male mice. Group symbols and color codes are indicated. Statistical analysis was performed using the two‐way ANOVA followed by Tukey's post hoc test for body weight or temperature changes to compare PBS with each infected group. *p < 0.05; **p < 0.01; ***p < 0.001; **** *p* < 0.0001. **Supporting Information Figure 3.** JEV‐specific IgG titers before genotype 3 or 5 cross‐challenge. Five‐week‐old C57BL/6 mice (*n* = 4 per group) were immunized with PBS (negative control), JYJEV3, or JYJEV5. Serum was collected at the indicated time points. These groups were later challenged as described in Figure 5. (A) JEV3‐specific IgG titers determined by ELISA. (B) JEV5‐specific IgG titers determined by ELISA. Data represent mean ± SD. *p < 0.05, and **p < 0.01. **Supporting Information Figure 4.** Construction of reporter gene‐expressing pBAC JYJEV5 plasmids. Schematic diagram showing the strategy for generating pBAC‐JYJEV5‐NLuc and PBAC‐JYJEV5‐EGFP. A reporter cassette (5′ UTR‐C38‐reporter gene‐FMDV‐2A‐scrambled capsid‐prME) was assembled via overlapping PCR and inserted using In‐Fusion cloning. Primer sequences are listed in Supplementary Table 4. **Supporting Information Figure 5.** Genetic stability of the JYJEV5‐NLuc reporter virus. (A‐C) JYJEV5‐NLuc was serially passaged in Vero cells (P1–P10). (A) RT‐PCR analysis using primers spanning the 5′ UTR to the capsid region. (B) Wild‐type JEV G5 (lacking the NLuc) served as a negative control. Red arrow, full‐length reporter cassette; blue arrow, truncated form. (C) NLuc activity measured at 72 hpi using a plate reader. All data are presented as mean values from at least three independent experiments. Data represent mean ± SD. ****p < 0.0001. M, marker; N, non‐template control; WT, wild‐type virus. **Supporting Information Figure 6.** Genetic stability of the JYJEV5‐EGFP reporter virus. (A‐D) JYJEV5‐EGFP was serially passaged in Vero cells (P1–P10). (A) RT‐PCR with primers spanning the 5′ UTR to the capsid region. (B) Wild‐type JEV G5 (lacking the EGFP) was used as a control. Red arrow, full‐length reporter; blue arrow, truncated form. (C) EGFP fluorescence visualized by microscopy at 72 hpi. (D) Fluorescence intensity quantified via plate reader. All data are presented as mean values from at least three independent experiments. M, marker; N, non‐template control; WT, wild‐type virus. **Supporting Information Figure 7**. Correlation between reporter gene expression and viral titers in JYJEV5 reporter virus‐infected Vero cells. (A) Linear regression analysis of NLuc luminescence intensity versus viral titers (PFU/mL) in the supernatants of Vero cells infected with JYJEV5‐based‐NLuc. (B) Linear regression analysis of EGFP fluorescence intensity versus viral titers (PFU/mL) in the supernatants of Vero cells infected with JYJEV5‐based‐EGFP. Reporter signal values were plotted against corresponding viral titers determined by plaque assay, measured at 24 h intervals post‐infection. The regression line and *R*² values were calculated using GraphPad Prism. Data represent at least three independent experiments. **Supporting Table 1.** List of primers used to amplify JEV G5 genome fragments. **Supporting Table 2.** List of primers used for pBAC‐JYJEV5 Construction. **Supporting Table 3.** Sequence differences between the parent strain and JYJEV5 arising unintentionally during clone assembly. **Supporting Table 4.** List of primers used for the construction of pBAC‐JYJEV5 reporter gene. **Supporting Data 1. Supporting Data 2. Supporting Data 3**.

## Data Availability

The data that supports the findings of this study are available in the Supporting Information S1: Supporting Material of this article.
