# Supplementary material for: Japanese Encephalitis Virus Genotype 5 Infectious Clone and Reporter System for Antiviral Evaluation
Source: J Med Virol. 2025 Sep 16;97(9):e70608. doi: 10.1002/jmv.70608 (PMC12439625; doi:10.1002/jmv.70608)
Supplement: Supplementary file 1 — Supporting Information Figure 1. Construction of the pBAC‐JYJEV5 infectious clone. (A) Schematic representation of the pBAC‐JYJEV5 construct comprising three fragments. (B) PCR amplification of each fragment was resolved on an agarose gel. (C) Agarose gel electrophoresis confirmed PCR amplification of each fragment. M, marker; N, non‐template control; F1, Fragment 1; F2, Fragment 2; F3, fragment 3. Supporting Information Figure 2. Physiological responses following intracerebral inoculation with JYJEV3 and JYJEV5. (A) Schematic representation of experimental design. Male and Female C57BL/6 mice (n = 5 per group) were intracerebrally injected with PBS, JYJEV3 (250 PFU), or JYJEV5 (2000 PFU) at a dose corresponding to 70‐fold of the LD50 and monitored for 12 days. (B and C) Changes in body weight (B) and body temperature (C) in female mice. (D and E) Changes in body weight (D) and body temperature (E) in male mice. Group symbols and color codes are indicated. Statistical analysis was performed using the two‐way ANOVA followed by Tukey's post hoc test for body weight or temperature changes to compare PBS with each infected group. *p < 0.05; **p < 0.01; ***p < 0.001; **** p < 0.0001. Supporting Information Figure 3. JEV‐specific IgG titers before genotype 3 or 5 cross‐challenge. Five‐week‐old C57BL/6 mice (n = 4 per group) were immunized with PBS (negative control), JYJEV3, or JYJEV5. Serum was collected at the indicated time points. These groups were later challenged as described in Figure 5. (A) JEV3‐specific IgG titers determined by ELISA. (B) JEV5‐specific IgG titers determined by ELISA. Data represent mean ± SD. *p < 0.05, and **p < 0.01. Supporting Information Figure 4. Construction of reporter gene‐expressing pBAC JYJEV5 plasmids. Schematic diagram showing the strategy for generating pBAC‐JYJEV5‐NLuc and PBAC‐JYJEV5‐EGFP. A reporter cassette (5′ UTR‐C38‐reporter gene‐FMDV‐2A‐scrambled capsid‐prME) was assembled via overlapping PCR and inserted using In‐Fusion clon [file JMV-97-e70608-s001.docx]

**Supporting Information**

**Japanese Encephalitis Virus Genotype 5 Infectious Clone and Reporter System for Antiviral Evaluation**

Jae-Yeon Park^1^, Wataru Kamitani^2^, Hyun-Jin Shin^1*^, Hye-Mi Lee^1*^

^1^ College of Veterinary Medicine, Chungnam National University, Daejeon, South Korea; ^2^ Department of Infectious Diseases and Host Defense, Gunma University Graduate School of Medicine, Gunma, Japan

*Correspondences: hyelee0728@cnu.ac.kr (Hye-Mi Lee), shin0089@cnu.ac.kr (Hyun-Jin Shin)

**Supplementary Materials and methods**

***Plaque assay for quantification of JYJEV5 and reporter viruses***

To quantify the parental and recombinant viruses (JYJEV5, JYJEV5-NLuc, and JYJEV5-EGFP), plaque assays were performed using Vero cells. Cells were seeded in 12-well plates to 80–90% confluency and infected with either JYJEV5 or reporter viruses. All virus samples were serially diluted 10-fold in MEM. After removing the growth medium, 200 μL of each virus dilution was added to the wells and incubated for 1 h at 37℃ in a CO_2_ incubator. The inoculum was then removed, and the cells were overlaid with 1.5% carboxymethyl cellulose (Wako, Japan) prepared in MEM containing 5% FBS and 1**×** Antibiotic-Antimycotic. Plates were incubated for 5 days at 37°C under 5% CO₂. After incubation, the overlay medium was removed, and cells were fixed with 4% paraformaldehyde in PBS for 30 min at RT. Fixed cells were stained with 0.5% crystal violet solution in 20% methanol for 15 min. Plaques were scanned and counted using a CTL ELISpot reader (Cellular Technology Limited, OH, USA).

***Cyro-EM of parental and recombinant virus***

Cryo-EM was performed to visualize the morphology of the parental and recombinant virus. Virus sample (3 µL) were applied to glow-discharged holey carbon grids (Quantifoil R1.2/1.3 Au300; Quantifoil Micro Tools GmbH) and vitrified using a Vitrobot Mark IV (Thermo Fisher Scientific, MA, USA) at 4°C under 100% humidity. Grid images were obtained using a Talos Arctica G2 transmission electron microscope (Thermo Fisher Scientific), operated at 200 kV in parallel illumination mode at the Korea Basic Science Institute. Micrographs were acquired in EFTEM mode using a 20 eV slit width with a BioQuantum energy filter and a K3 direct electron detector (Gatan Inc., CA, USA) at a nominal magnification of 72,000×, corresponding to a pixel size of 1.7 Å, with spot size 3 and a 50 µm C2 aperture. Data were collected using EPU software (Thermo Fisher Scientific) in electron counting mode for 5.0 s at a defocus value of -3.5 µm, with a dose rate of 18.6 electrons per pixel per second, resulting in a cumulative dose of 50 electrons per Å².

***Monitoring of body weight and temperature in JEV-infected mice***

To assess disease progression following infection, body weight and body temperature were monitored daily for 12 days post-infection. Eight-week-old male and female C57BL/6 mice were divided into six experimental groups (n=5 per group): PBS controls, JYJEV3, and JYJEV5 groups for each sex. Mice were intracerebrally inoculated with 70-fold the LD₅₀ of either JYJEV3 (250 PFU) or JYJEV5 (2000 PFU). Body temperature was measured daily using a rectal thermometer probe (RET-3, used with BAT-12 thermometer, Physitemp Instruments Inc., Clifton, NJ, USA) at the same time each day to minimize circadian variation. The probe was inserted more than 2 cm into the rectum to obtain colonic temperature, which serves as an estimate of core body temperature [1]. Infection resulted in weight loss and disease manifestation, including hunched posture and ruffled fur. Mice exhibiting ≥ 20% body weight loss or signs of severe neurological impairment were euthanized using CO₂ asphyxiation. Changes in body weight and temperature were compared with those of the PBS control group. Statistical analysis was performed using two-way ANOVA followed by Tukey’s post hoc test in GraphPad Prism Version 8.0.

***ELISA for quantification of virus-specific IgG in immunized mouse sera***

ELISA quantified virus-specific IgG titers in the sera of immunized mice. Briefly, 96-well plates were coated overnight at 4℃ with purified parental virus (JEV3 and JEV5) and subsequently blocked with 3% skim milk for 30 min at RT. Serially diluted mouse serum samples were added to the wells and incubated for 2 h at RT. After washing three times with PBSt (PBS containing 0.1% Triton X-100), the plates were incubated with horseradish peroxidase (HRP)-conjugated goat-anti-mouse IgG antibody (Cusabio, CSB-PA477109, China). The signal was developed using TMB substrate (Labis Koma, South Korea), and the reaction was terminated by the addition of 2N H_2_SO_4_ (Labis Koma). Absorbance was measured at 450 nm using a VICTOR Nivo microplate reader (PerkinElmer).

***SN assay using sera from immunized mice***

SN assays were performed to determine the titers of virus-neutralizing antibodies. Sera from immunized samples were heat-inactivated at 56°C for 30 min and subjected to 2-fold serial dilutions. Each diluted sample was mixed with an equal volume of JEV3 or JEV5 (200 TCID_50_/0.05 mL) and incubated for 1 h at 37°C. Vero cells were infected with 0.1 mL of the virus-serum mixture and incubated for 1 h at 37°C. After infection, the cells were washed twice with PBS and maintained in MEM for 5 days. Each condition was tested in duplicate using 96-well plates. Neutralizing antibody titers were defined as the highest serum dilution that completely inhibited CPE.

***Quantification of viral RNA in mouse brain tissue by qRT-PCR***

To detect viral RNA in the brains of infected mice, total RNA was isolated using the Tissue RNA Extraction Kit (Geneall), following the manufacturer’s protocol. Briefly, brain tissues were homogenized in PBS at a fixed ratio (20 mg tissue per 1 mL), and equal volumes of the homogenates were subjected to RNA extraction to ensure consistency across samples. cDNA was synthesized from the purified RNA using TOPscript™ RT DryMIX (dT18) (Enzynomics). To detect viral RNA levels, results were expressed as genome copy numbers. For absolute quantification, standard curves were generated using viral RNA extracted from Vero cell-propagated wild-type JEV G3 and G5 virus stocks, with genome copy numbers calculated based on RNA concentrations corresponding to plaque-forming unit equivalents per milliliter (PFUeq/mL) as determined by plaque assays [2]. To detect viral RNA levels were expressed as genome copy numbers. For absolute quantification, standard curves were generated using viral RNA extracted from Vero cell-propagated wild-type JEV3 and JEV5 virus stocks, with genome copy numbers calculated based on TCID_50_-derived RNA concentrations. All samples were analyzed in duplicate.

qRT-PCR was performed using SFCgreen® I mixture (Biofact) on a QuantStudio™ 1 Real-Time PCR System (Thermo Fisher Scientific). The cycling conditions were 95°C for 10 min, followed by 40 cycles at 95°C for 30 s and 58°C for 30 s. The qRT-PCR primers used were JEV3 forward: 5′-AGACCAACGTCAGGCCACAA-3′ and reverse: 5′-TCTACAGCTT

CGAGGGGGCT-3′, and JEV5 forward: 5′-GACCGGGTAACCAAAGCCGA-3′ and reverse: 5′-ATTTCGGCGCTCTGTGCCTT-3′.

***Cytokine and Chemokine Expression Profiling by qRT-PCR in Mouse Brain***

Whole brains were harvested, and total RNA was extracted from homogenized brain tissues using the AllEx® Mini Cell/Tissue Total RNA Kit (GeneAll) according to the manufacturer’s instructions for the AllEx® Mini Automated Nucleic Acid Extraction System (GeneAll). cDNA synthesis was performed using the TOPscript™ RT DryMIX (dT18; Enzynomics). qRT-PCR was conducted using SYBR Green PCR Master Mix (Enzynomics) on a QuantStudio™ 1 Real-Time PCR System (Thermo Fisher Scientific). Amplification was carried out for 40 cycles under the following conditions: 95°C for 10 sec and 60°C for 30 sec. Gene expression levels were calculated using the 2(-^ΔΔ^Ct) method, *Gapdh* serving as the internal control gene. Results are presented as relative fold changes compared to the PBS group. Primer sequences used for qRT-PCR were as follows: *Tnf* forward: 5′-CAGGCGGTGCCTATGTCTC-3’ and reverse: 5’-CGATCACCCCGAAGTTCAGTAG-3’, *Il6* forward: 5’-GAGGATACCACTCCCAACAGAC C-3’ and reverse: 5’-AAGTGCATCATCGTTGTTCATACA-3’, *Ifng* forward: 5’-TCAAGTGG

CATAGATGTGGAAGAA-3’ and reverse: 5’-TGGCTCTGCAGGATTTTCATG-3’, *Cxcl10* forward: 5’-CCAAGTGCTGCCGTCATTTTC-3’ and reverse: 5’-GGCTCGCAGGGATGAT

TTCAA-3’, *Ccl5* forward: 5’-CTGCTGCTTTGCCTACCTCTC-3’ and reverse: 5’-CTTGCA

GGGGTCAGGATAGT-3’ *Ccl4* forward: 5’-TGCTCGCATCATCCGACTG-3’ and 5’-GCTGCTGGTCTTGGTTAGGC-3’, and *Gapdh* forward: 5’-TGTGTCCGTCGTGGATCTG

A-3’ and reverse: 5’-CCTGCTTCACCACCTTCTTGA-3’

***Cytokine Expression by Western Blot Analysis in Mouse Brain***

Whole mouse brains were harvested, and total protein extraction, brain tissues were homogenized in RIPA buffer (Thermo Fisher Scientific) supplemented with protease and phosphatase inhibitor cocktails (Sigma-Aldrich). Homogenates were incubated on ice for 30 minutes and centrifuged at 12,000 × g for 15 minutes at 4°C. The supernatants were collected, and total protein concentrations were determined using the BCA Protein Assay Kit (Thermo Fisher Scientific). Equal amounts of protein were separated on 10% SDS-PAGE gels and transferred onto PVDF membranes (Millipore, MA, USA). Membranes were blocked with 5% non-fat dry milk in TBS-T (Tris-buffered saline with 0.1% Tween-20) for 1 h at RT and incubated overnight at 4°C with the following primary antibodies: anti-TNF (Cell Signaling Technology, MA, USA) and anti-IL-6 (Cell Signaling Technology). After washing, membranes were incubated with HRP-conjugated secondary antibodies (Cell Signaling Technology) for 2 h at RT. Signals were visualized using SuperSignal West Femto Maximum Sensitivity Substrate ECL solution (Thermo Fisher Scientific) and imaged with an ATTO Luminograph (ATTO corporation, Japan). β-actin (Santa Cruz Biotechnology, CA, USA) was used as a loading control.

***Assessment of Reporter Gene Stability by RT-PCR***

To examine the genetic stability of the JYJEV5-NLuc and JYJEV5-EGFP, viral RNA was extracted at each passage was extracted using the Ribospin vRD kit (GeneAll South Korea), followed by cDNA synthesis using the TOPscript™ RT DryMIX dT18/dN6 plus (Enzynomics, South Korea). The regions spanning from 5’UTR to C gene encompassing the NLuc or EGFP insertion was amplified using specific primers: JEV G5 5’UTR-Forward (5’-GCAGTTTAAACAGTTTTTTAGAACGGAAGAAAACC-3’) and JEV G5 C-Reverse (5’-TGCACTCACACTGGCAACCACAACTGCCAAGCCC-3’). Additionally, amplicons were cloned into the pGEM-T Easy vector and analyzed by Sanger sequencing.

**Reference**

1. C.W. Meyer, Y. Ootsuka, and A.A. Romanovsky. "Body Temperature Measurements for Metabolic Phenotyping in Mice,"*Frontiers in Physiology* 8 (2017): 520.

2. S.I. de la Cruz-Hernandez, H. Flores-Aguilar, S. Gonzalez-Mateos, et al. "Determination of viremia and concentration of circulating nonstructural protein 1 in patients infected with dengue virus in Mexico,"*American Journal of Tropical Medicine and Hygiene* 88 (2013): 446-454.

**
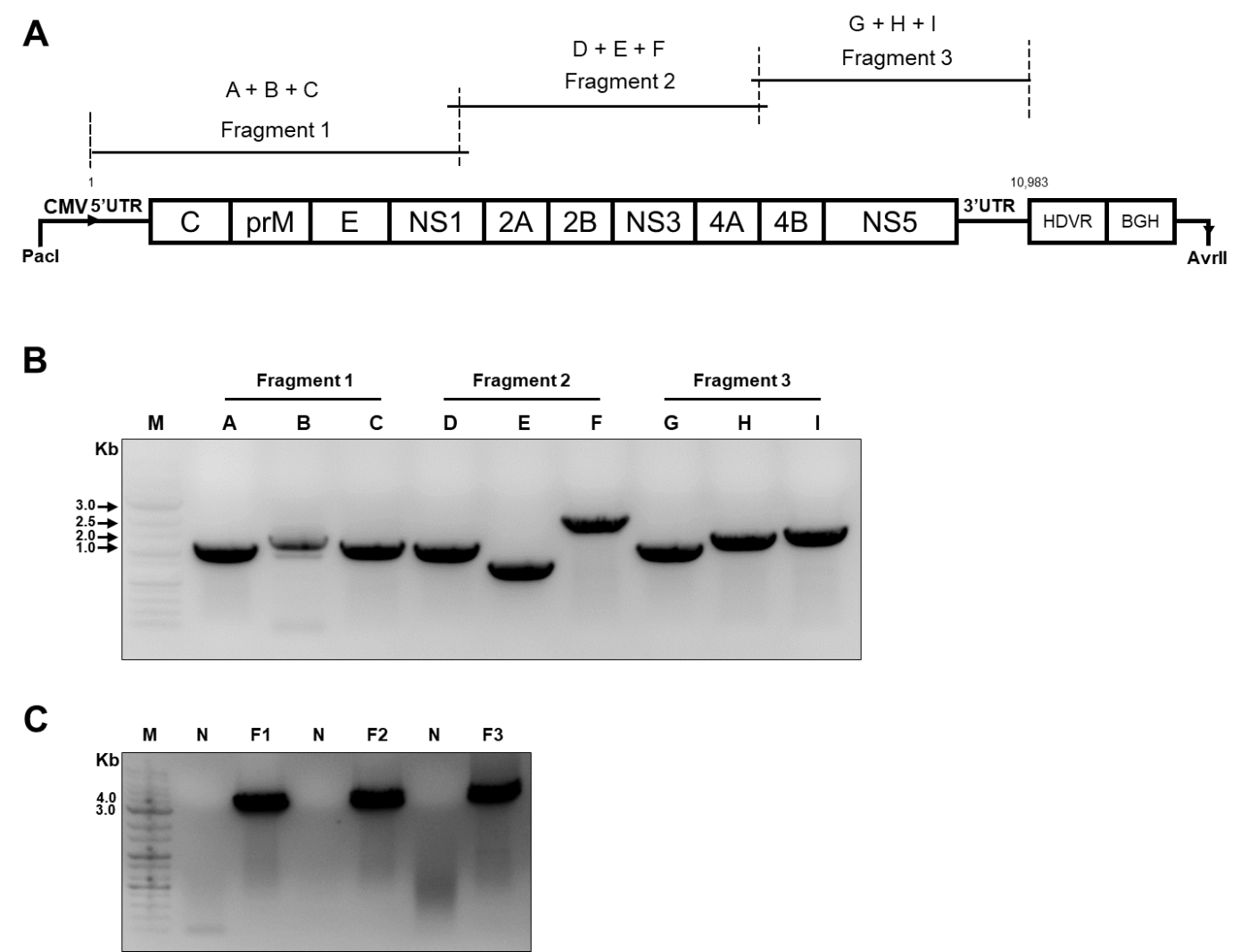
Supporting Information Figure 1.** Construction of the pBAC-JYJEV5 infectious clone. (A) Schematic representation of the pBAC-JYJEV5 construct comprising three fragments. (B) PCR amplification of each fragment was resolved on an agarose gel. (C) Agarose gel electrophoresis confirmed PCR amplification of each fragment. M, marker; N, non-template control; F1, Fragment 1; F2, Fragment 2; F3, fragment 3.

**
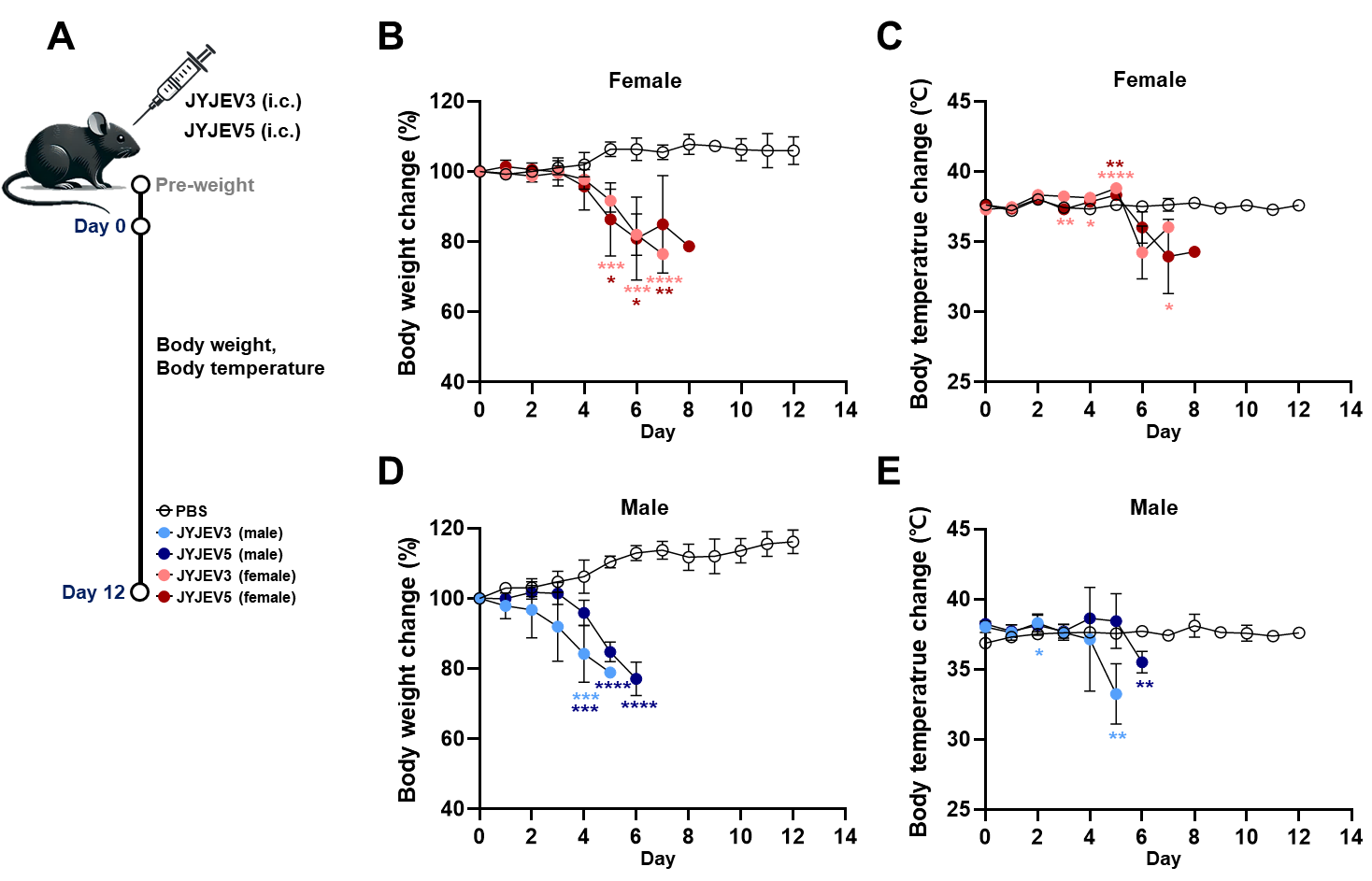
 Supporting Information Figure 2.** Physiological responses following intracerebral inoculation with JYJEV3 and JYJEV5. (A) Schematic representation of experimental design. Male and Female C57BL/6 mice (n = 5 per group) were intracerebrally injected with PBS, JYJEV3 (250 PFU), or JYJEV5 (2000 PFU) at a dose corresponding to 70-fold of the LD_50_ and monitored for 12 days. (B, C) Changes in body weight (B) and body temperature (C) in female mice. (D, E) Changes in body weight (D) and body temperature (E) in male mice. Group symbols and color codes are indicated. Statistical analysis was performed using the two-way ANOVA followed by Tukey’s post hoc test for body weight or temperature changes to compare PBS with each infected group. **p* < 0.05; ***p* < 0.01; ****p* < 0.001; **** *p* < 0.0001.

**
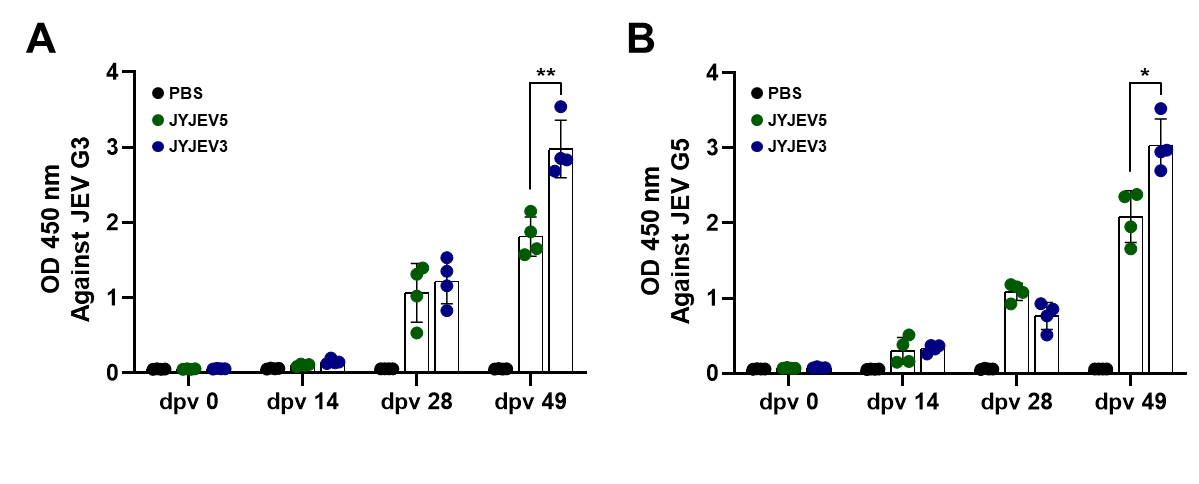
**

**Supporting Information Figure 3.** JEV-specific IgG titers before genotype 3 or 5 cross-challenge. Five-week-old C57BL/6 mice (n = 4 per group) were immunized with PBS (negative control), JYJEV3, or JYJEV5. Serum was collected at the indicated time points. These groups were later challenged as described in Figure 5. (A) JEV3-specific IgG titers determined by ELISA. (B) JEV5-specific IgG titers determined by ELISA. Data represent mean ± SD. **p* < 0.05, and ***p* < 0.01.


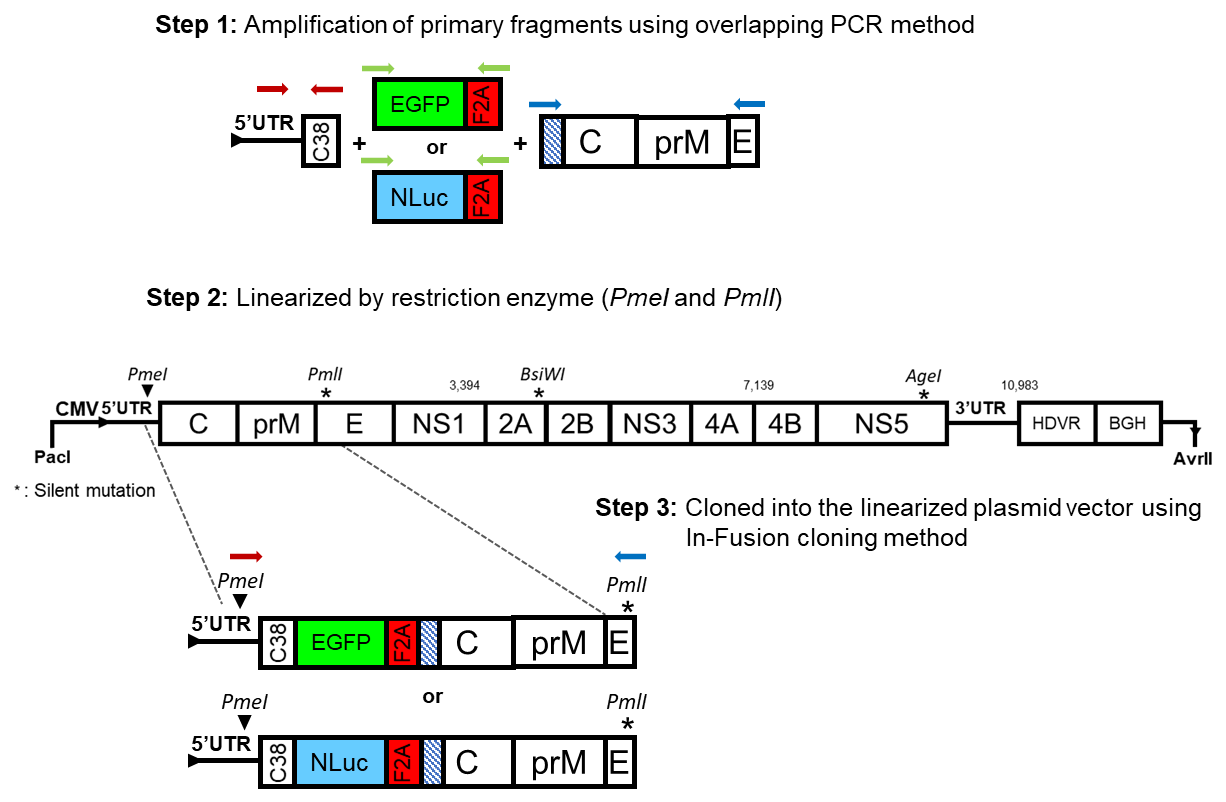


**Supporting Information Figure 4.** Construction of reporter gene-expressing pBAC JYJEV5 plasmids. Schematic diagram showing the strategy for generating pBAC-JYJEV5-NLuc and PBAC-JYJEV5-EGFP. A reporter cassette (5′ UTR-C38-reporter gene-FMDV-2A-scrambled capsid-prME) was assembled via overlapping PCR and inserted using In-Fusion cloning. Primer sequences are listed in Supplementary Table 4.

**
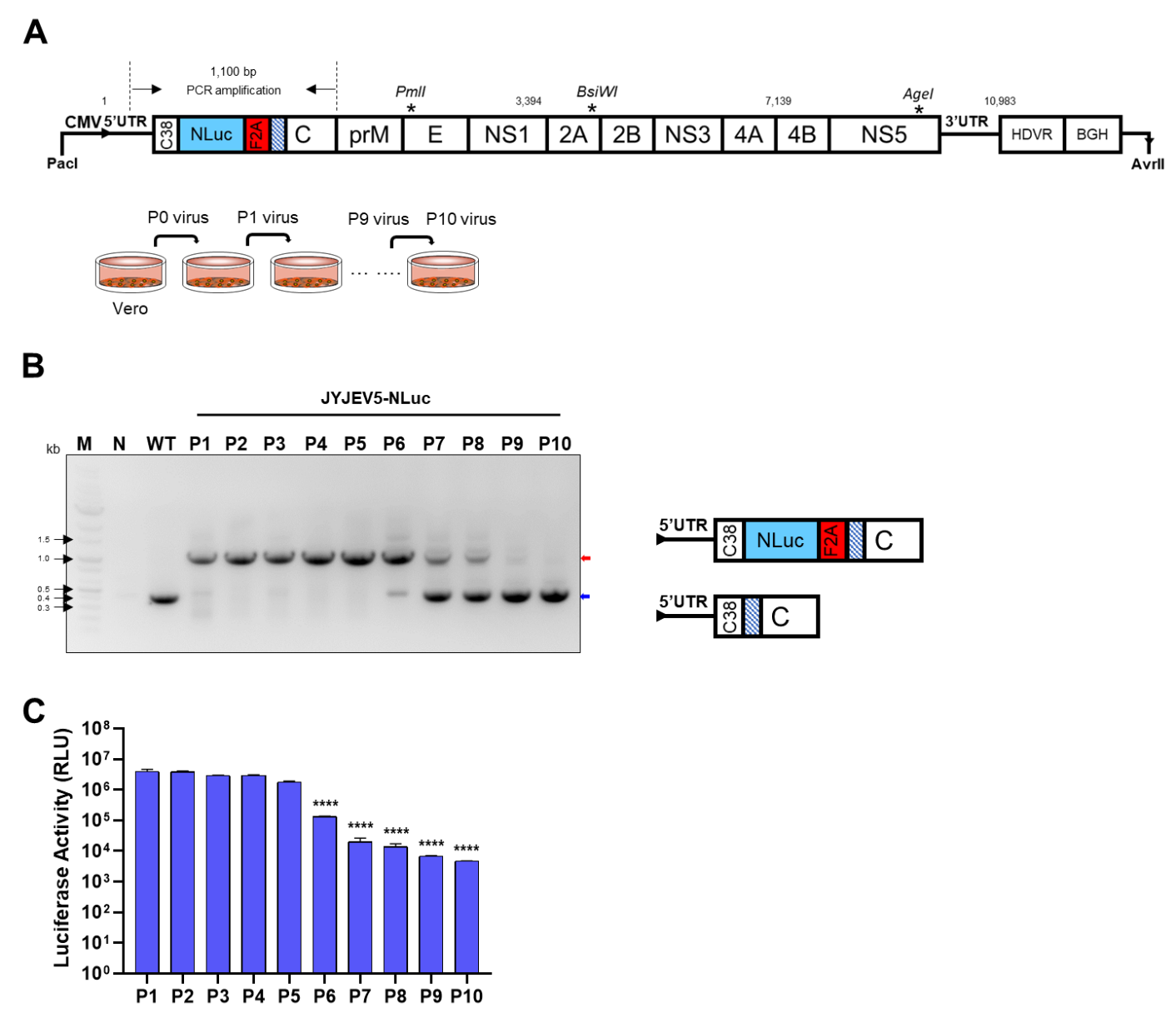
**

**Supporting Information Figure 5.** Genetic stability of the JYJEV5-NLuc reporter virus. (A-C) JYJEV5-NLuc was serially passaged in Vero cells (P1–P10). (A) RT-PCR analysis using primers spanning the 5′ UTR to the capsid region. (B) Wild-type JEV G5 (lacking the NLuc) served as a negative control. Red arrow, full-length reporter cassette; blue arrow, truncated form. (C) NLuc activity measured at 72 hpi using a plate reader. All data are presented as mean values from at least three independent experiments. Data represent mean ± SD. *****p* < 0.0001. M, marker; N, non-template control; WT, wild-type virus.


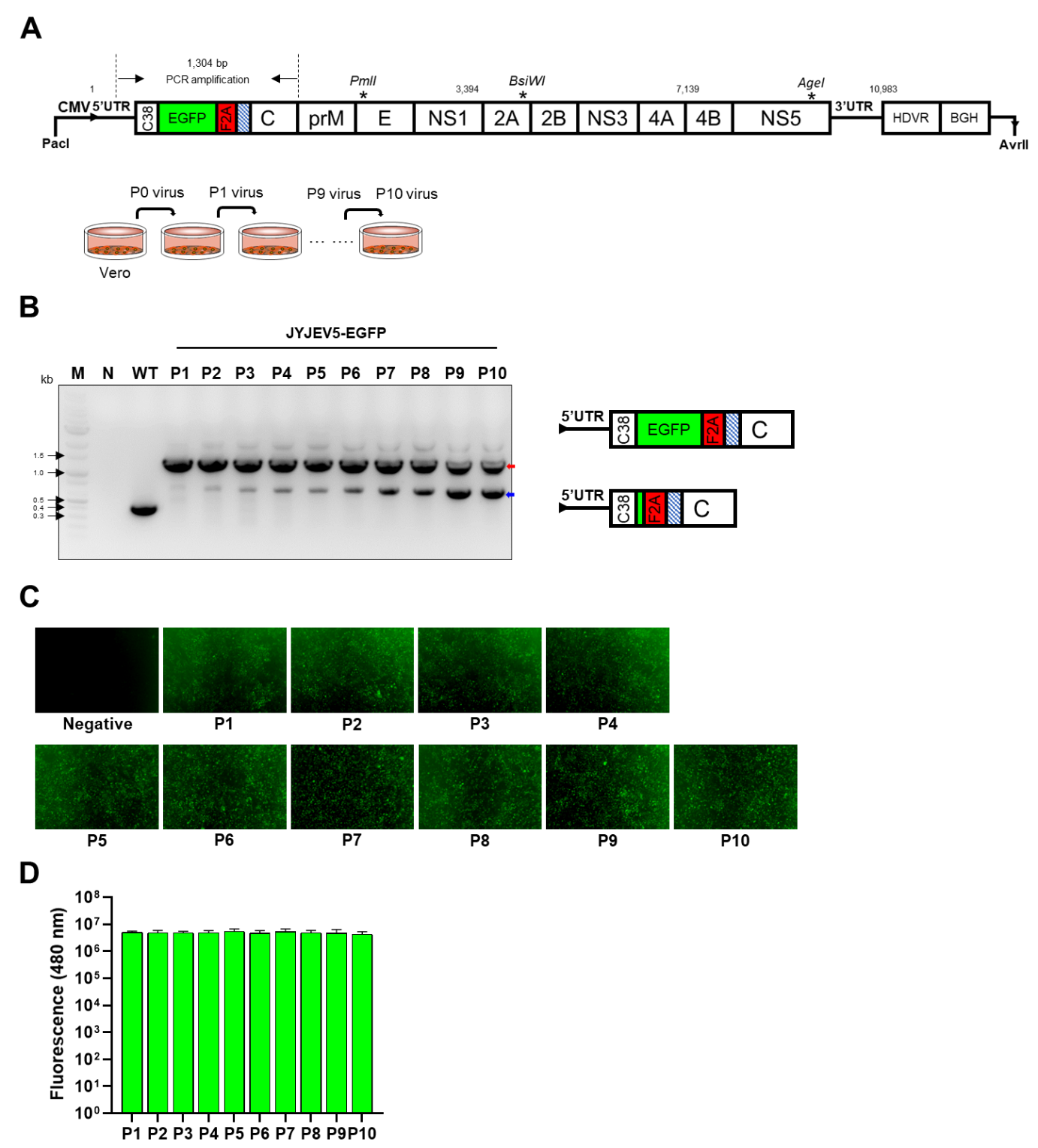


**Supporting Information Figure 6.** Genetic stability of the JYJEV5-EGFP reporter virus. (A-D) JYJEV5-EGFP was serially passaged in Vero cells (P1–P10). (A) RT-PCR with primers spanning the 5′ UTR to the capsid region. (B) Wild-type JEV G5 (lacking the EGFP) was used as a control. Red arrow, full-length reporter; blue arrow, truncated form. (C) EGFP fluorescence visualized by microscopy at 72 hpi. (D) Fluorescence intensity quantified via plate reader. All data are presented as mean values from at least three independent experiments. M, marker; N, non-template control; WT, wild-type virus.


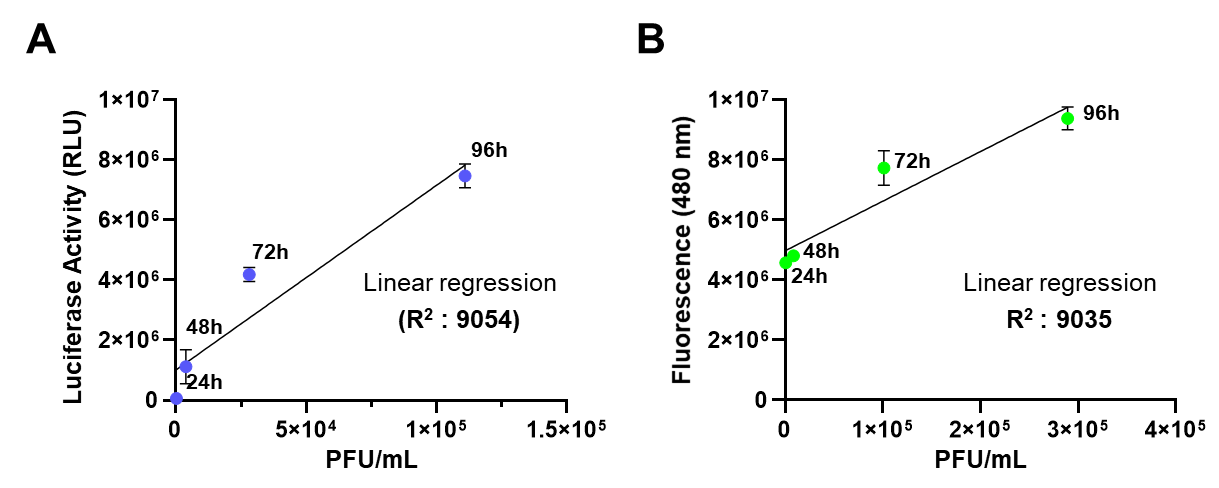


**Supporting Information Figure 7**. Correlation between reporter gene expression and viral titers in JYJEV5 reporter virus-infected Vero cells. (A) Linear regression analysis of NLuc luminescence intensity versus viral titers (PFU/mL) in the supernatants of Vero cells infected with JYJEV5-based-NLuc. (B) Linear regression analysis of EGFP fluorescence intensity versus viral titers (PFU/mL) in the supernatants of Vero cells infected with JYJEV5-based-EGFP. Reporter signal values were plotted against corresponding viral titers determined by plaque assay, measured at 24 h intervals post-infection. The regression line and R² values were calculated using GraphPad Prism. Data represent at least three independent experiments.

**Supplementary Tables**

**Supplementary Table 1.** List of primers used to amplify JEV G5 genome fragments.

**Supplementary Table 2.** List of primers used for pBAC-JYJEV5 Construction.


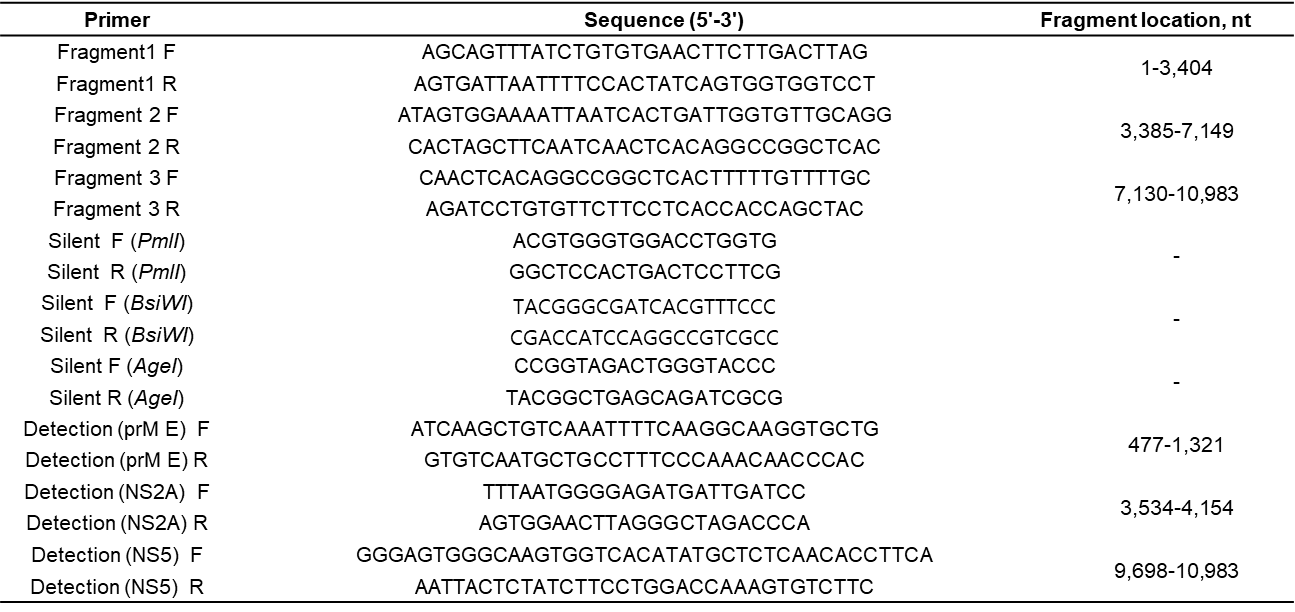


**Supplementary Table 3.** Sequence differences between the parent strain and JYJEV5 arising unintentionally during clone assembly.

**
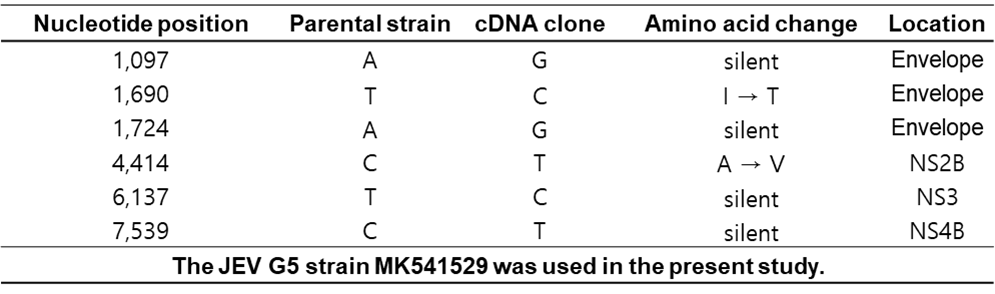
**

**Supplementary Table 4.** List of primers used for the construction of pBAC-JYJEV5 reporter gene.

**
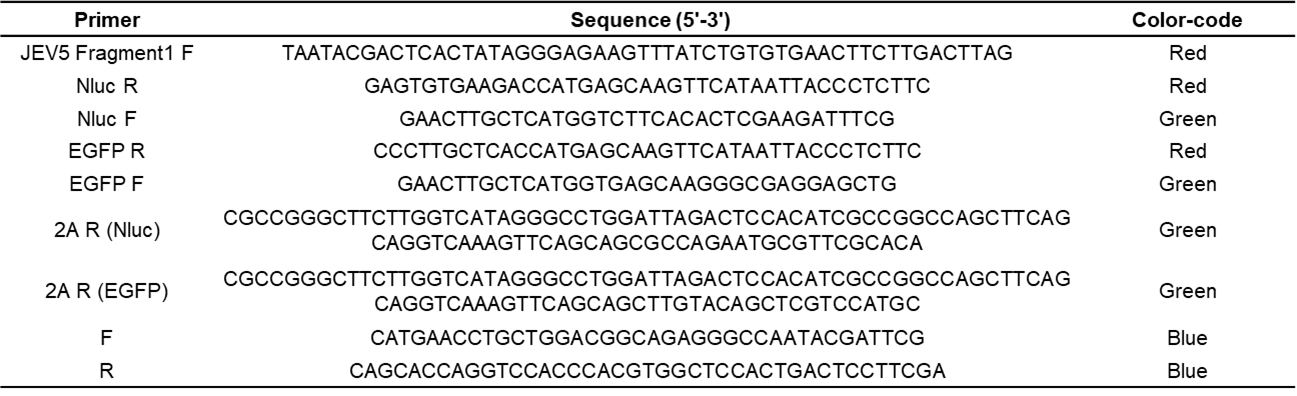
**

**Supplementary Data**

**Supplementary Data 1.** Complete sequence of JYJEV5.

The sequence of the viral cDNA, HDVr, BGH poly(A), and CMV promoter is shown in different colors. Nucleotides that differ from the parental sequence are indicated in lowercase and bold red font, and deliberately introduced mutations are underlined. This sequence was obtained from a virus stock at passage 20, and no additional mutations were detected compared to the original (P0 or pBAC JYJEV5) sequence.

5’UTR

AGCAGTTTATCTGTGTGAACTTCTTGACTTAGTATTGTTGAGAGGAATCGAGAGATTAGTGCAGTTTAAACAGTTTTTTAGAACGGAAGAAAACC

Capsid

ATGACTAAAAAACCAGGAGGGCCCGGTAAAAACCGGGCTATCAATATGCTGAAACGCGGCATACCCCGCGTATCCCCACTTGTGGGGGTGAAGAGGGTAATTATGAACTTGCTCGACGGCAGAGGGCCAATACGATTCGTTTTGGCTCTCTTGGCGTTTTTCAAGTTCACAGCACTAGCCCCGACCAAGGCACTCGTTAGCCGATGGAAGGCAGTAGAGAAGAGCGTTGCAATGAAACATCTCACCAGTTTCAAAAAGGAACTGGGAACGCTCATCAACGCTGTAAATAAGAGGGGCAAAAAACAAAACAAAAGAGGAGGAAGTAATGGGACAGTCATCTGGATAATGGGCTTGGCAGTTGTGGTTGCCAGTGTGAGTGCA

prMembrane

ATCAAGCTGTCAAATTTTCAAGGCAAGGTGCTGATGACAATCAACAACACCGATGTGGCTGATGTGATCACCATTCCCACCTCAAAAGGGACTAATAGATGTTGGGTCCGAGCTATAGATGTGGGACACATGTGCGAGGACACAATCACTTATGAATGCCCAAAGCTTGACGCTGGCAACGACCCGGAGGACATTGACTGCTGGTGCGACAAACAAGCCGTGTATGTCCAGTATGGACGTTGCACTAGGACCAGGCACTCCAGGAGAAGTAGAAGATCTGTGTCAGTGCAAACCCACGGAGAAAGTTCTCTAGTGAACAAAAAAGAAGCCTGGATGGATTCGACGAAAGCCACTCGGTATCTCATGAAAACAGAGAATTGGATCATACGGAATCCAGGCTATGCTCTTGTGGCAGTGGCACTTGGATGGATGCTTGGTAGCAACAACGGCCAGCGTGTGGTATTCACAATTCTCTTGCTGTTGGTCGCACCCGCATATAGC

Envelope

TTCAACTGCTTAGGTATGGGAAATCGTGACTTCATCGAAGGAGTCAGTGGAGC**c**ACGTGGGTGGACCTGGTGCTGGAAGGAGACAGTTGCCTCACCATCATGGCGAACGACAAACCAAC**g**TTGGACGTGCGCATGATAAACATCGAGGCCACGGAACTGGCTGAGGTGCGAACCTACTGCTACCACGCCACAGTGGCTGACATCTCAACAGTGGCAAGATGCCCCACGACTGGAGAAGCCCATAACACGAAGCGAGCTGACAGCAGCTATGTCTGCAAGCAAGGCTACACTGATCGTGGATGGGGAAACGGATGTGGGTTGTTTGGGAAAGGCAGCATTGACACATGCGCTAAATTTGTTTGCAGCCACAAGGCCATTGGAAAGATAATACAGCCAGAAAACATCAAATATGAAGTTGGAGTGTTTGTTCATGGAACCACAACAGCAGAGAACCACGGAAATTACACAGCCCAGATTGGGGCTTCCCAGGCTGCCAAGTTCACCATCACGCCCAATGCTCCTTCCATCACTCTGAAGCTTGGAGATTACGGAGAAGTCACGATGGATTGCGAGCCGCGTAGTGGATTTAATACTGAGGCATTTTACGTGCTGACCGTAGGGTCTAAGTCGTTCCTAGTCCACCGCGAATGGTTCAATGATCTTGCGCTTCCGTGGCTATCTCCATCTAGCACAAATTGGAGAAATAGAGAGA**c**CCTGATGGAGTTCGAAGAGGCTCACGCGACAAA**g**CAGTCTGTCGTCGCGCTTGGATCACAAGAGGGAGCCTTGCATCAGGCATTGGCTGGTGCCATAGTGGTGGAGTACTCTAGCTCAGTGAAGTTGACATCTGGTCACCTCAAATGCAGACTAAAAATGGAGAAGTTGGCGTTGAAAGGGACCACCTATGGTATGTGCACAGAGAAGTTCTCTTTTTCCAAGAATCCAGCTGACACTGGTCATGGTACGGTTGTCATAGAATTGCAGTACACCGGCACTGACGGACCTTGCAAGATACCCATTTCTTCGGTGGCCAGTCTGAATGATTTAACTCCAGTTGGTAGATTGGTGACAGTCAATCCTTTTGTTGCCACATCCACCGCCAATTCGAAGGTTTTGGTAGAATTGGAACCACCATTTGGAGATTCATTCATTGTTGTCGGAAGAGGAGATAAGCAGATCAATCACCATTGGCACAAGGCTGGCAGTTCACTGGGAAAGGCTTTCACGACTACTTTGAAAGGAGCTCAGAGGCTGGCAGCCCTTGGTGACACCGCTTGGGACTTTGGATCCATTGGAGGGGTTTTCAATTCCATTGGTAAGGCCGTGCACCAAGTGTTTGGAGGAGCTTTCAGAACCCTTTTTGGTGGCATGTCCTGGATAACACAAGGATTGATGGGAGCGCTGCTGCTGTGGATGGGTATCAACGCACGAGATCGGTCGATTGCACTAGCTTTTCTTGCTACAGGAGGTGTGCTCTTGTTTCTGGCCACCAATGTCCACGCC

NS1

GATACCGGCTGCGCCATCGATATAACCAGAAAAGAAATGAGGTGTGGTAGTGGCATATTTGTGCACAATGACGTGGAGGCTTGGGTTGATAGATACAAGTATCTACCTGAGACTCCCAAGTCTTTAGCCAAAATAGTCCACAAAGCACACAAGGAAGGCATTTGTGGAGTGAGATCAGTCACCAGACTGGAACACCAAATGTGGGAGGCCGTCAGGGACGAGTTAAATGTCTTGTTGAAGGAGAATGCGGTAGATCTTAGTGTGGTGGTGGACAAACCATCAGGAAGATACCGACCAGCTCCATTGCGGTTGGCCATGACTCAGGAAAAGTTTGAGATGGGTTGGAAAGCATGGGGGAAGAGCATTCTCTTTGCGCCGGAACTGGCCAATTCCACGTTTGTGATCGACGGACCTGAAACCAAAGAGTGTCCAGATGAGCGTAGAGCATGGAACAGCATGCAGATTGAGGATTTTGGGTTTGGCATTACGTCGACTCGAGTGTGGTTGAAGATCAGGGAGGAGCGCACGAATGAATGTGATGGAGCCATCATCGGCACGGCTGTTAAAGGGAACATGGCAGTGCACAGTGACTTGTCATACTGGATTGAAAGCCATCTCAACGACACCTGGAAGCTCGAGAGAGCTGTGTTTGGAGAGATAAAGTCTTGCACTTGGCCAGAAACACACACGCTCTGGGGAGATGGTGTTGAGGAAAGTGAGTTGATAATTCCACACACACTCGCTGGACCCAAAAGCAAGCATAACAGAAGAGAGGGTTACAAGACACAGAATCAGGGACCATGGGACGAGAGTGAGATCACTCTTGATTTTGACTACTGTCCAGGGACCACAGTCACCATTGCTGAGGGATGTGGGAAAAGGGGGCCCTCAATCAGGACCACCACTGATAGTGGAAAATTAATCACTGATTGGTGTTGCAGGAGCTGCACTTTGCCGCCACTGAGGTTCAGGACAGCCAGTGGGTGCTGGTATGGAATGGAAATACGGCCCATGAAGCATGATGAATCCACGCTCGTGAAATCACAAGTCAACGCG

NS2A

TTTAATGGGGAGATGATTGATCCTTTTCAGTTGGGCCTTCTGGTGATTTTTCTGGCCACCCAGGAGGTCCTTCGCAAGAGGTGGACGGCCAGACTGACGATTCCTGCGGTTTTGGGGGCCCTACTTGTTCTGATGCTTGGGGGCATCACCTACACTGATCTAGTGAGATATGTGGTGTTGGTGGCTGCTGCTTTCGCTGAAGCTAACAATGGAGGCGATGTAGTTCATTTGGCCCTGATTGCCGTATTTAAAATCCAACCGGCATTCCTAGTCATGAGCATAGCAAGAACAAATTGGACAAACCAGGAAAACATTGCCCTAGTGCTAGGAGCTGCTTTCTTTCAGATGGCCTCAACAGACCTGGAGTTTGGTATCCATGGGCTGCTAAATGCAGCGGCGACGGCCTGGATGGT**c**GT**a**CGGGCGATCACGTTTCCCACGACCTCCACCATCACAATGCCCATACTAGCTTTGCTGGCGCCAGGAATGAGAGCTCTCCATCTTGACACCTACAGAATCTTTTTGCTTATCATCGGGGTCTGTGCTCTGCTGCATGAAAGGAGGAAAACCATGGCGAAGAAGAAAGGTGCTGTCCTTTTGGGTCTAGCCCTAAGTTCCACT

NS2B

GGATGGCCAGCGACAGAATTTCTGTCTGCGATTGGGTTGATGTTTGCTATTGTTGGGGGCCTGGCCGAGTTGGACATCGACTCTATGGCGATACCTTTCATGTTAGCTGGGCTTATGGCAGTGTCATATGTGGTATCAGGAAAAGCAACGGACATGTGGCTAGAACGTGCGGCCGACATTAGTTGGGAAGTGGATGCAG**t**GATCACAGGCAGCAGCCGAAGGTTGGACGTCAAACTAGATGATGATGGAGATTTCCACCTTATTGATGATCCAGGCGTCCCATGGAAAATTTGGGTACTGCGCATGTCTTGCATAGGATTGGCGGCCTTCACACCATGGGCCATCATCCCAGCGGCTTTTGGATATTGGCTGACTTTGAAAACCACAAAGAGA

NS3

GGAGGCGTTTTTTGGGACACACCATCACCCAAAGTCTACGCAAAAGGGGACACAACCACAGGAGTGTACAGGATAATGGCTCGAGGGATCTTTGGCGTCTACCAAGCAGGTGTTGGAGTGATGTATGAGAACGTGTTCCACACTCTGTGGCACACGACCAGAGGAGCTGCCATAATGAGTGGAGAAGGAAAGTTGACGCCGTACTGGGGAAGCGTTAAGGAGGACCGCATAACTTATGGGGGCCCATGGAGATTCGATCGAAAATGGAATGGAGTGGATGACGTGCAAATGATTGTAGTTGAACCAGGAAAAGCAGCTGTAAATGTCCAAACAAAGCCGGGAGTGTTTCGGACTCCACACGGAGAAATTGGAGCTGTTAGCCTGGATTACCCTAGTGGGACATCGGGCTCACCCATCCTGGACATCAACGGTGATATCATTGGATTGTATGGGAACGGAGTTGAACTTGGAGATGGCTCATATGTGAGCGCCATTGTGCAGGGTGAACGGCAAGAAGAGCCCATCCCCGATGCATACAATCCAAACATGCTCAAGAAAAGGCAGCTGACCGTGTTGGACTTGCATCCAGGATCTGGGAAAACAAGGAAAATCTTACCCCAAATCATCAAGGATGCCATTCAACAGCGTCTCAGAACAGCTGTTCTGGCGCCCACTCGGGTTGTTGCAGCTGAGATGGCAGAAGCTCTTAGAGGACTTCCCGTTAGATACCAAACTTCAGCGGTTCCGCGAGAACATCAGGGGAATGAGATAGTTGATGTCATGTGTCACGCCACCTTAACGCATAAGCTGATGTCACCAAATCGCGTCCCCAATTACAACTTGTTTGTCATGGATGAGGCCCATTTCACAGACCCAGCCAGCATCGCCGCTAGAGGATACATATCCACCAGAGTGGAATTGGGAGAAGCCGCGGCTATCTTCATGACTGCCACTCCACCAGGAACGACTGACCCCTTCCCTGACTCCAACGCTCCCATTCATGATTTGCAGGACGAGATCCCTGACAGAGCATGGAGCAGTGGGTATGAATGGATAACTGAGTACTCTGGGAAGACAGTATGGTTTGTGGCGAGTGTGAAAATGGGCAACGAAATCGCAGTGTGTCTACAGAGAGCCGGAAAGAGAGTCATCCAGTTAAATCGGAAATCTTATGACACCGAGTACCCCAAATGTAAGAATGGGGATTGGGATTTTGTCATCACCACGGACATTTCTGAAATGGGGGCCAACTTTGGAGCGAGCAGAGTGATTGATTGTAGGAAGAGTGTGAAACCCACCATTCTGGAAGAAGGAGAAGGAAGAGTCATTCTCAGCAACCCATCGCCTATCACCAGTGCGAGTGCAGCCCAGCGGAGAGGCAGAGTGGGCAGAAATCCAAATCAGGTTGGAGATGAGTACCATTACGGAGGGGTCACAAGTGAAGATGACACCAACCTAGCACACTGGACAGAAGCCAAGATCATGTTGGACAACATCCACCTGCCAAATGGGTTGGTAGCTCAGCTCTA**c**GGACCTGAAAGGGAAAAGGCCTTCACAATGGACGGTGAGTATCGATTGAGGGGTGAAGAAAAGAAGAACTTTCTGGAGTTAATCAGAACAGCCGACCTCCCCGTATGGCTAGCTTACAAAGTGGCTTCAAATGGAATACAGTACACCGATAGGAGATGGTGTTTTGATGGACCTCGGACGAATGCTATCTTAGAAGACAGCACTGAAGTAGAGATAATCACCAGAACGGGAGAAAGGAAAATTCTAAAACCAAGATGGCTGGACGCACGGGTGTACGCAGATCACCAGGCTTTGAAGTGGTTTAAGGACTTCGCAGCAGGAAAGAGA

NS4A

TCAGCTGTCAGTTTTCTAGAGGTGCTTGGGCGCATGCCAGAGCACTTCATGGGGAAAACGCGTGAAGCTCTTGATACAATGTACCTGGTTGCCACGGCAGAAAAAGGGGGAAAAGCTCACCGAATGGCCCTGGAGGAACTGCCAGATGCACTGGAGACGGTGACACTCATCGCAGCGCTTGCCGTGATGACAGGTGGGTTCTTTCTACTCATGATGCAACGAAAGGGCATAGGAAAAATGGGCCTCGGGGCTCTCGTGCTCACCCTGGCCACTTTCTTCTTGTGGATGGCAGAGGTCTCAGGGACGAAAATAGCCGGGACCTTACTCATAGCGTTGCTGCTTATGGTGGTACTCATCCCGGAGCCGGAGAAGCAAAGATCCCAAACGGACAACCAGTTGGCCGTGTTTCTGATCTGCGTCCTCACCGTAGTGGGAATTGTGGCTGCT

NS4B

AATGAGTATGGCATGCTCGAAAAGACCAAGGAAGACATAAGGAATGTGTTTGGTAACAAGGTTCAGACATCCAATGCACCTGGAAGCCTATCAAGTCTGGCGCTCGATCTACGACCAGCAACGGCTTGGGCCTTATATGGAGGTAGTACAGTGATTTTAACTCCACTGCTGAAACATTTGATCACCTCTGAGTATGTGACAACATCACTAGCTTCAATCAACTCACAGGCCGGCTCACTTTTTGTTTTGCCAAAAGGCATGCCTTTCACGGATTTGGATCTGACGGTTGGACTCGTCTTTCTGGGTTGTTGGGGGCAAATCACTCTCACCACTTTTCTGACAGCTGGAGTGTTAGTAGTTCTGCATTACGGCTATATGCTCCCTGGCTGGCAAGCTGAAGCCCTGAGGGCAGCTCAGAGACGAACAGCTGCGGGTATCATGAAGAACGCCGTTGTGGATGGGATGGTTGCCACTGATGTGCCTGAATTGGAAAGAACAACACCCCTAATGCAGAAGAAGGTGGGGCAAGTGTTGCTAATAGGAGTCAGTATAGCAGCTTTTCTTGTCAATCCCAATGTCACCACCGTGCGAGAAGCCGGTGTGCTGGTGACCGCTGCTACGCTCACCtTATGGGATAATGGAGCAAGTGCCGTCTGGAATTCAACTACAGCCACAGGACTCTGCCACGTCATGCGAGGCAGTTACTTGGCTGGTGGTTCAATAGCTTGGACTCTCATCAAGAATGTTGATAAACCATCCTTGAAAAGA

NS5

GGAAGGCCTGGAGGAAGAACGCTGGGTGAGCAATGGAAAGAAAAATTGAACGCCATGAACAAAGAAGAGTTTTTCAGGTACAGGAAAGAAGCCATAGTTGAGGTGGACCGCACAGAGGCACGCAGGGCTAGACGAGAGAATAACAAAGTGGGAGGTCATCCCGTGTCACGAGGTTCAGCAAAGCTCCGATGGATGGTGGAGAAAGGGTTTGTCTCCCCTGTTGGAAAGGTTGTGGACCTTGGCTGTGGGCGGGGAGGATGGTGCTATTATGCTGCCACTCTGAAAAAAGTGCAAGAAGTTAAAGGTTATACAAAAGGAGGGGCCGGACATGAGGAACCGATGCTGATGCAAAGCTATGGTTGGAATCTGGTCACGATGAAGAGTGGAGTGGATGTGTTCTACAGACCTTCAGAGCCTAGTGACACCCTGCTCTGTGACATAGGGGAGTCTTCTCCAAGTCCAGACGTCGAGGAGCAACGCACTCTGCGGGTTCTGGAAATGGCATCAGAGTGGCTACACCGAGGACCCAGAGAATTCTGCATTAAAGTTTTGTGTCCATACATGCCAAAGGTGATAGAAAAGATGGAAACGCTACAACGTCGCTTTGGAGGCGGACTGGTGCGCGTTCCCCTGTCACGCAACTCAAATCACGAAATGTACTGGGTTAGTGGGGCTGCTGGGAACGTGGTACATGCTGTAAACATGACTAGTCAAGTTTTGCTGGGGCGAATGGACCGACCAGTCTGGAGAGGACCCAAATATGAAGAAGATGTCAACTTGGGAAGCGGGACCAGAGCTGTAGGGAAAGGTGAGGTTCACAGTGACCAAGGAAAAATCAAGAAGCGGATAGAGAAGCTGAAAGAAGAGTATGCAGCGACGTGGCACGAGGACCCTGAACATCCATACCGCACTTGGACATACCATGGAAGCTATGAAGTGAAGGCCACCGGTTCAGCCAGCTCCCTTGTCAACGGAGTGGTTAAGCTCATGAGTAAACCTTGGGATGCCATCACTAGTGTCACCACCATGGCCATGACTGACACTACTCCCTTTGGTCAGCAGAGAGTCTTCAAAGAAAAAGTTGACACCAAGGCGCCTGAGCCACCTGCAGGAGTTCGGGAAGTGCTGGACGAAACTACCAACTGGCTGTGGGCCTACTTGTCAAGAGAGAAAAGACCTCGCTTGTGTACGAGAGAGGAGTTCATCCGGAAAGTCAACAGCAACGCAGCTCTTGGAGCCATGTTTGCCGAGCAAAATCAGTGGAGCTCAGCCAGGGAGGCTGTTAGTGACCCGGCCTTCTGGAACATGGTCGACATTGAAAGAGAGAACCACCTACGAGGGGAGTGCCACACCTGCATCTACAACATGATGGGAAAAAGAGAAAAGAAACCTGGTGAGTTTGGGAAGGCTAAAGGAAGCAGAGCTATCTGGTTCATGTGGCTCGGAGCCCGCTACCTGGAATTCGAGGCACTCGGGTTCCTGAACGAGGACCATTGGCTGAGCAGGGAGAACTCTGGAGGAGGAGTGGAAGGCTCAGGCATACAGAAGCTAGGGTACATTTTGCGAGACATCTCAACGAAATCTGGAGGGAAAATGTATGCTGATGACACCGCAGGCTGGGACACTAGGATCACAAGGGTTGATCTGGACAATGAGGCAAAGGTGCTGGAACTTCTGGATGGGGAGCACAGGATGTTGGCCCGTGCTATTATAGAATTGACTTACAAACACAAAGTTGTCAAAGTAATGAGGCCAGCAGTAGGTGGAAAGACCGTGATGGATGTGATCTCTAGAGAAGATCAAAGAGGGAGTGGGCAAGTGGTCACATATGCTCTCAACACCTTCACAAACATAGCTGTCCAGCTAGTGAGGTTGATGGAGGCTGAAGGGGTTGTCGGTCCACAGGATGTGGAGCAGCTCCCCAGGAAAACCAAATTCGCAGTCAGGACATGGCTTTTTGAAAATGGAGAAGAGAGAGTCACCAGAATGGCAGTGAGTGGGGATGACTGCGTTGTCAAACCTTTGGATGACAGATTCGCGCATGCTTTACACTTCTTGAATGCGATGTCAAAGGTGAGGAAAGACATACAAGAATGGAAGCCATCTCAAGGCTGGCACGACTGGCAGCAAGTCCCTTTCTGCTCGAACCACTTTCAGGAGATTGTGATGAAGGATGGTAGAAGCCTTGTTGTGCCCTGCCGGGGACAGGATGAGTTAATAGGCAGGGCCCGGATTTCACCAGGAGCAGGATGGAATGTGAGAGACACAGCCTGCCTAGCTAAGGCCTACGCCCAAATGTGGCTCCTCCTCTACTTTCACCGGAGAGACTTGCGCCTTATGGCCAACGCGATCTGCTCAGCCGT**a**CCGGTAGACTGGGTACCCACAGGTAGGACATCATGGTCGATACACTCAAAAGGAGAGTGGATGACAACAGAAGACATGCTGCAGGTGTGGAACAGGGTATGGATTGAGGAGAATGAATGGATGAGAGACAAAACCCCCGTCGCCAGTTGGACCGACGTCCCATACGTCGGAAAGAGGGAAGACATCTGGTGCGGCAGCTTGATCGGAACGCGGACAAGGGCCACCTGGGCAGAAAACATCTATGCAGCAATAAACCAAGTGAGAGCAATAATTGGAAATGAAAAGTATGTGGACTACATGACATCACTTAGGAGGTATGAAGACACTTTGGTCCAGGAAGATAGAGTAATTTAA

3’UTR

AAAACTTTTGGTAATGAGTGTAAATAGTAGTATTTATTGTAAATAGTGTAAATAAACAAATTTAAATAGGAAGTCAGGCCGACGTAAGTCGCCACCGGATGCTGAGTAGACGGTGCTGCCTGCGTCTCAGCCCCAGGAGGACTGGGTTAACAAATCTGACAACCGAAGGTAGGAAAGCCCTCAAAACCGTCTCGGAAGAAGGTCCCTGCTTACTGGAGGTTGGAAGACCGTGTCAGGCCACGCAAGTGCCACTTCGCTGAGGAGTGCAGCCTGTACAGCCCCGGGAGGACCGGGTAACCAAAGCCGATGAGGCCCCCACGGCCCAAACCTCATCCAGGATGCAATGGATGAGGCGTAAGGACTAGAGGTTAGAGGAGACCCCGTGGAAAAGAAAATGCGGCCCAAACTCTTTCGAAGCTGTAGAAGGAGTGGAAGGACTAGAGGTTAGAGGAGACCCCGCATTTGCATCAAAACAGCATATTGACACCTGGATTAGACTAGGAGATCTTCTGATCTATCTCAACATCAGCTACAAGGCACAGAGCGCCGAAATATGTAGCTGGTGGTGAGGAAGAACACAGGATCT

HDVr

GGGTCGGCATGGCATCTCCACCTCCTCGCGGTCCGACCTGGGCATCCGAAGGAGGACGCACGTCCACTCGGATGGCTAAGGGAGAGCCAGCCT

BGH

CGACTGTGCCTTCTAGTTGCCAGCCATCTGTTGTTTGCCCCTCCCCCGTGCCTTCCTTGACCCTGGAAGGTGCCACTCCCACTGTCCTTTCCTAATAAAATGAGGAAATTGCATCGCATTGTCTGAGTAGG TGTCATTCTATTCTGGGGGGTGGGGTGGGGCAGGACAGCAAGGGGGAGGATTGGGAAGACAATAGCAGGCATGCTGGGGATGCGGTGGGCTCTATGG

CMV promoter

CGTTACATAACTTACGGTAAATGGCCCGCCTGGCTGACCGCCCAACGACCCCCGCCCATTGACGTCAATAATGACGTATGTTCCCATAGTAACGCCAATAGGGACTTTCCATTGACGTCAATGGGTGGAGTATTTACGGTAAACTGCCCACTTGGCAGTACATCAAGTGTATCATATGCCAAGTACGCCCCCTATTGACGTCAATGACGGTAAATGGCCCGCCTGGCATTATGCCCAGTACATGACCTTATGGGACTTTCCTACTTGGCAGTACATCTACGTATTAGTCATCGCTATTACCATGGTGATGCGGTTTTGGCAGTACATCAATGGGCGTGGATAGCGGTTTGACTCACGGGGATTTCCAAGTCTCCACCCCATTGACGTCAATGGGAGTTTGTTTTGGCACCAAAATCAACGGGACTTTCCAAAATGTCGTAACAACTCCGCCCCATTGACGCAAATGGGCGGTAGGCGTGTACGGTGGGAGGTCTATATAAGCAGAGCT

Nanoluciferase

ATGGTCTTCACACTCGAAGATTTCGTTGGGGACTGGCGACAGACAGCCGGCTACAACCTGGACCAAGTCCTTGAACAGGGAGGTGTGTCCAGTTTGTTTCAGAATCTCGGGGTGTCCGTAACTCCGATCCAAAGGATTGTCCTGAGCGGTGAAAATGGGCTGAAGATCGACATCCATGTCATCATCCCGTATGAAGGTCTGAGCGGCGACCAAATGGGCCAGATCGAAAAAATTTTTAAGGTGGTGTACCCTGTGGATGATCATCACTTTAAGGTGATCCTGCACTATGGCACACTGGTAATCGACGGGGTTACGCCGAACATGATCGACTATTTCGGACGGCCGTATGAAGGCATCGCCGTGTTCGACGGCAAAAAGATCACTGTAACAGGGACCCTGTGGAACGGCAACAAAATTATCGACGAGCGCCTGATCAACCCCGACGGCTCCCTGCTGTTCCGAGTAACCATCAACGGAGTGACCGGCTGGCGGCTGTGCGAACGCATTCTGGCG

EGFP

ATGGTGAGCAAGGGCGAGGAGCTGTTCACCGGGGTGGTGCCCATCCTGGTCGAGCTGGACGGCGACGTAAACGGCCACAAGTTCAGCGTGTCCGGCGAGGGCGAGGGCGATGCCACCTACGGCAAGCTGACCCTGAAGTTCATCTGCACCACCGGCAAGCTGCCCGTGCCCTGGCCCACCCTCGTGACCACCCTGACCTACGGCGTGCAGTGCTTCAGCCGCTACCCCGACCACATGAAGCAGCACGACTTCTTCAAGTCCGCCATGCCCGAAGGCTACGTCCAGGAGCGCACCATCTTCTTCAAGGACGACGGCAACTACAAGACCCGCGCCGAGGTGAAGTTCGAGGGCGACACCCTGGTGAACCGCATCGAGCTGAAGGGCATCGACTTCAAGGAGGACGGCAACATCCTGGGGCACAAGCTGGAGTACAACTACAACAGCCACAACGTCTATATCATGGCCGACAAGCAGAAGAACGGCATCAAGGTGAACTTCAAGATCCGCCACAACATCGAGGACGGCAGCGTGCAGCTCGCCGACCACTACCAGCAGAACACCCCCATCGGCGACGGCCCCGTGCTGCTGCCCGACAACCACTACCTGAGCACCCAGTCCGCCCTGAGCAAAGACCCCAACGAGAAGCGCGATCACATGGTCCTGCTGGAGTTCGTGACCGCCGCCGGGATCACTCTCGGCATGGACGAGCTGTACAAG

FMDV-2A

CTGCTGAACTTTGACCTGCTGAAGCTGGCCGGCGATGTGGAGTCTAATCCAGGCCCT

Capsid (with scrambled C38)

ATGACCAAGAAGCCCGGCGGCCCCGGCAAGAACAGAGCCATCAACATGCTGAAGAGAGGCATCCCTAGAGTGAGCCCCCTGGTGGGCGTGAAGAGAGTGATCATGAACCTGCTGGACGGCAGAGGGCCAATACGATTCGTTTTGGCTCTCTTGGCGTTTTTCAAGTTCACAGCACTAGCCCCGACCAAGGCACTCGTTAGCCGATGGAAGGCAGTAGAGAAGAGCGTTGCAATGAAACATCTCACCAGTTTCAAAAAGGAACTGGGAACGCTCATCAACGCTGTAAATAAGAGGGGCAAAAAACAAAACAAAAGAGGAGGAAGTAATGGGACAGTCATCTGGATAATGGGCTTGGCAGTTGTGGTTGCCAGTGTGAGTGCA

**Supplementary Data 2.** Sequence detail corresponding to PCR bands in the Supporting Information Figure 5B.


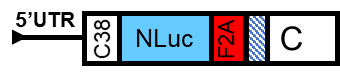
The expected amplicon from JYJEV5-NLuc encompasses the 5′UTR through the C gene. The corresponding nucleotide sequence of this PCR product is shown in Supporting Information Figure 5B. The start codon is indicated in bold red, while the Nluc and FMDV-2A sequences are color coded to match the schematic illustration (upper). The scrambled capsid region is highlighted in italic bold to distinguish it from the native sequence.

**Upper band (1160 bp), corresponding to reporter-positive amplicon from JYJEV5-NLuc.**

AGCAGTTTATCTGTGTGAACTTCTTGACTTAGTATTGTTGAGAGGAATCGAGAGATTAGTGCAGTTTAAACAGTTTTTTAGAACGGAAGAAAACC**ATG**ACTAAAAAACCAGGAGGGCCCGGTAAAAACCGGGCTATCAATATGCTGAAACGCGGCATACCCCGCGTATCCCCACTTGTGGGGGTGAAGAGGGTAATTATGAACTTGCTCATGGTCTTCACACTCGAAGATTTCGTTGGGGACTGGCGACAGACAGCCGGCTACAACCTGGACCAAGTCCTTGAACAGGGAGGTGTGTCCAGTTTGTTTCAGAATCTCGGGGTGTCCGTAACTCCGATCCAAAGGATTGTCCTGAGCGGTGAAAATGGGCTGAAGATCGACATCCATGTCATCATCCCGTATGAAGGTCTGAGCGGCGACCAAATGGGCCAGATCGAAAAAATTTTTAAGGTGGTGTACCCTGTGGATGATCATCACTTTAAGGTGATCCTGCACTATGGCACACTGGTAATCGACGGGGTTACGCCGAACATGATCGACTATTTCGGACGGCCGTATGAAGGCATCGCCGTGTTCGACGGCAAAAAGATCACTGTAACAGGGACCCTGTGGAACGGCAACAAAATTATCGACGAGCGCCTGATCAACCCCGACGGCTCCCTGCTGTTCCGAGTAACCATCAACGGAGTGACCGGCTGGCGGCTGTGCGAACGCATTCTGGCGCTGCTGAACTTTGACCTGCTGAAGCTGGCCGGCGATGTGGAGTCTAATCCAGGCCCT***ATGACCAAGAAGCCCGGCGGCCCCGGCAAGAACAGAGCCATCAACATGCTGAAGAGAGGCATCCCTAGAGTGAGCCCCCTGGTGGGCGTGAAGAGAGTGATCATGAACCTGCTG***GACGGCAGAGGGCCAATACGATTCGTTTTGGCTCTCTTGGCGTTTTTCAAGTTCACAGCACTAGCCCCGACCAAGGCACTCGTTAGCCGATGGAAGGCAGTAGAGAAGAGCGTTGCAATGAAACATCTCACCAGTTTCAAAAAGGAACTGGGAACGCTCATCAACGCTGTAAATAAGAGGGGCAAAAAACAAAACAAAAGAGGAGGAAGTAATGGGACAGTCATCTGGATAATGGGCTTGGCAGTTGTGGTTGCCAGTGTGAGTGCA

**Lower band (515 bp), corresponding to reporter-positive amplicon from JYJEV5-NLuc.**


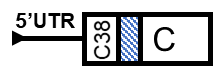


AGCAGTTTATCTGTGTGAACTTCTTGACTTAGTATTGTTGAGAGGAATCGAGAGATTAGTGCAGTTTAAACAGTTTTTTAGAACGGAAGAAAACC**ATG**AATAAAAAACCAGGAGGGCCCGGTAAAACCCGGGATATCAATATGATGAAACGCGGCATACCCCGCGTATCCCCACTTTTGGGGGTGAAGAGGGTAATTAGGAACT***TGAGCCCCCTGGTGGGCGTGAAGAGAGTGATCATGAACCTGCTG***GACGGCAGAGGGCCAATACGATTCGTTTTGGCTCTCTTGGCGTTTTTCAAGTTCACAGCACTAGCCCCGACCAAGGCACTCGTTAGCCGATGGAAGGCAGTAGAGAAGAGCGTTGCAATGAAACATCTCACCAGTTTCAAAAAGGAACTGGGAACGCTCATCAACGCTGTAAATAAGAGGGGCAAAAAACAAAACAAAAGAGGAGGAAGTAATGGGACAGTCATCTGGATAATGGGCTTGGCAGTTGTGGTTGCCAGTGTGAGTGCA

**Supplementary Data 3.** Sequence detail corresponding to PCR bands in the Supporting Information Figure 6B.

The expected amplicon from JYJEV5-EGFP encompasses the 5′UTR through the C gene. The corresponding nucleotide sequence of this PCR product is shown in Supporting Information Figure 6B. The start codon is indicated in bold red, while the EGFP and FMDV-2A sequences are color coded to match the schematic illustration (upper). The scrambled capsid region is highlighted in italic bold to distinguish it from the native sequence.


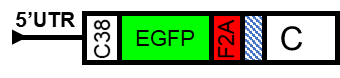
**Upper band (1364 bp), corresponding to reporter-positive amplicon from JYJEV5-EGFP.**

AGCAGTTTATCTGTGTGAACTTCTTGACTTAGTATTGTTGAGAGGAATCGAGAGATTAGTGCAGTTTAAACAGTTTTTTAGAACGGAAGAAAACC**ATG**ACTAAAAAACCAGGAGGGCCCGGTAAAAACCGGGCTATCAATATGCTGAAACGCGGCATACCCCGCGTATCCCCACTTGTGGGGGTGAAGAGGGTAATTATGAACTTGCTCATGGTGAGCAAGGGCGAGGAGCTGTTCACCGGGGTGGTGCCCATCCTGGTCGAGCTGGACGGCGACGTAAACGGCCACAAGTTCAGCGTGTCCGGCGAGGGCGAGGGCGATGCCACCTACGGCAAGCTGACCCTGAAGTTCATCTGCACCACCGGCAAGCTGCCCGTGCCCTGGCCCACCCTCGTGACCACCCTGACCTACGGCGTGCAGTGCTTCAGCCGCTACCCCGACCACATGAAGCAGCACGACTTCTTCAAGTCCGCCATGCCCGAAGGCTACGTCCAGGAGCGCACCATCTTCTTCAAGGACGACGGCAACTACAAGACCCGCGCCGAGGTGAAGTTCGAGGGCGACACCCTGGTGAACCGCATCGAGCTGAAGGGCATCGACTTCAAGGAGGACGGCAACATCCTGGGGCACAAGCTGGAGTACAACTACAACAGCCACAACGTCTATATCATGGCCGACAAGCAGAAGAACGGCATCAAGGTGAACTTCAAGATCCGCCACAACATCGAGGACGGCAGCGTGCAGCTCGCCGACCACTACCAGCAGAACACCCCCATCGGCGACGGCCCCGTGCTGCTGCCCGACAACCACTACCTGAGCACCCAGTCCGCCCTGAGCAAAGACCCCAACGAGAAGCGCGATCACATGGTCCTGCTGGAGTTCGTGACCGCCGCCGGGATCACTCTCGGCATGGACGAGCTGTACAAGCTGCTGAACTTTGACCTGCTGAAGCTGGCCGGCGATGTGGAGTCTAATCCAGGCCCT***ATGACCAAGAAGCCCGGCGGCCCCGGCAAGAACAGAGCCATCAACATGCTGAAGAGAGGCATCCCTAGAGTGAGCCCCCTGGTGGGCGTGAAGAGAGTGATCATGAACCTGCTG***GACGGCAGAGGGCCAATACGATTCGTTTTGGCTCTCTTGGCGTTTTTCAAGTTCACAGCACTAGCCCCGACCAAGGCACTCGTTAGCCGATGGAAGGCAGTAGAGAAGAGCGTTGCAATGAAACATCTCACCAGTTTCAAAAAGGAACTGGGAACGCTCATCAACGCTGTAAATAAGAGGGGCAAAAAACAAAACAAAAGAGGAGGAAGTAATGGGACAGTCATCTGGATAATGGGCTTGGCAGTTGTGGTTGCCAGTGTGAGTGCA

**Lower band (695 bp), corresponding to reporter-positive amplicon from JYJEV5-NLuc.**


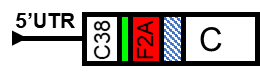


AGCAGTTTATCTGTGTGAACTTCTTGACTTAGTATTGTTGAGAGGAATCGAGAGATTAGTGCAGTTTAAACAGTTTTTTAGAACGGAAGAAAACC**ATG**ACTAAAAAACCAGGAGGGCCCGGTAAAAACCGGGCTATCAATATGCTGAAACGCGGCATACCCCGCGTATCCCCACTTGTGGGGGTGAAGAGGGTAATTATGAACTTGCTCATGGTGAGCAAGGGCGAGGAGCTGTTCACCGGGGTGGAGCTGTACAAGCTGCTGAACTTTGACCTGCTGAAGCTGGCCGGCGATGTGGAGTTTAATCCAGGCCCT***ATGACCAAGAAGCCCGGCGGCCCCGGCAAGAACAGAGCCATCAACATGCTGAAGAGAGGCATCCCTAGAGTGAGCCCCCTGGTGGGCGTGAAGAGAGTGATCATGAACCTGCTG***GACGGCAGAGGGCCAATACGATTCGTTTTGGCTCTCTTGGCGTTTTTCAAGTTCACAGCACTAGCCCCGACCAAGGCACTCGTTAGCCGATGGAAGGCAGTAGAGAAGAGCGTTGCAATGAAACATCTCACCAGTTTCAAAAAGGAACTGGGAACGCTCATCAACGCTGTAAATAAGAGGGGCAAAAAACAAAACAAAAGAGGAGGAAGTAATGGGACAGTCATCTGGATAATGGGCTTGGCAGTTGTGGTTGCCAGTGTGAGTGCA
